# CRISPR/Cas9 Mutagenesis by Translocation of Cas9 Protein Into Plant Cells via the *Agrobacterium* Type IV Secretion System

**DOI:** 10.3389/fgeed.2020.00006

**Published:** 2020-07-17

**Authors:** Daan J. Schmitz, Zahir Ali, Chenglong Wang, Fatimah Aljedaani, Paul J. J. Hooykaas, Magdy Mahfouz, Sylvia de Pater

**Affiliations:** ^1^Plant Sciences, Institute of Biology, Leiden University, Leiden, Netherlands; ^2^Division of Biological Sciences & Center for Desert Agriculture, King Abdullah University of Science and Technology, Thuwal, Saudi Arabia

**Keywords:** *Agrobacterium tumefaciens*, protein translocation, CRISPR/Cas, type 4 secretion system, genome editing, *Tobacco Rattle Virus* (TRV)

## Abstract

Clustered regularly interspaced short palindromic repeats (CRISPR)/CRISPR-associated protein 9 (Cas9) is a powerful tool for genome engineering in plants. The RNA-guided Cas9 endonuclease is usually delivered into plant cells as a DNA construct encoding Cas9 and the single guide RNA (sgRNA). However, constitutive expression of nucleases may cause off target mutations. In addition, DNA constructs can integrate into the host genome, causing mutations and complicating regulatory approval. Instead of DNA, here we deliver Cas9 through the *Agrobacterium* T4SS, accomplished by fusion of the VirF T4SS translocation peptide to Cas9 (NCas9F). Co-cultivation of Agrobacteria expressing NCas9F with yeast (*Saccharomyces cerevisiae*) harboring a sgRNA targeting *CAN1* showed that NCas9F was translocated via T4SS and induced targeted mutations in the yeast genome. Infiltration of *Nicotiana benthamiana* leaves with Agrobacteria expressing NCas9F and sgRNA-*PHYTOENE DESATURASE* (*PDS*) resulted in targeted modifications at the *PDS* locus, albeit at a very low rate. In order to increase the mutation frequency NCas9F protein was co-transported with a T-DNA encoding sgRNA-PDS1. Next generation sequencing confirmed that this resulted in targeted mutations at the *PDS* locus with a similar distribution but at a 5-fold lower frequency as the mutations obtained with a T-DNA encoding both Cas9 and sgRNA-PDS1. Similarly, infection with *Tobacco rattle virus* (TRV) encoding sgRNA-PDS2 combined with NCas9F protein translocation resulted in an equally high frequency of *PDS* mutations in *N. benthamiana* compared to T-DNA encoded sgRNA-PDS1 combined with NCas9F protein translocation. Our results revealed that translocation of NCas9F protein via the *Agrobacterium* T4SS can be used for targeted mutagenesis in host cells instead of the permanent and constitutive expression of Cas9 from a T-DNA.

## Introduction

Genome editing in plants can be achieved by the induction of site-specific double-stranded breaks (DSBs) in the genome. In recent years, the clustered regularly interspaced short palindromic repeats (CRISPR)/CRISPR-associated protein 9 (Cas9) system has become an important tool for generating targeted DNA DSBs and genetic modification of plant genomes. For targeted mutagenesis in plant cells, genes encoding the RNA-guided endonuclease CRISPR/Cas9 system are usually cloned onto a transfer DNA (T-DNA) and introduced into plant cells using *Agrobacterium tumefaciens*. *Agrobacterium*-mediated transformation stably integrates the T-DNA in the plant genome where it constitutively expresses the CRISPR/Cas9 machinery for targeted DNA modifications. Translocation of the T-DNA occurs via the type IV secretion system (T4SS), encoded by the *virB* genes and the *virD4* gene on the *Agrobacterium* Ti plasmid (Christie and Cascales, 2005; Alvarez-Martinez and Christie, 2009).

Although expression of the CRISPR/Cas9 system from a construct integrated in the host genome can effectively be used to engineer targeted mutations, constitutive expression may cause off-target mutations (Zhang et al., 2018; Hahn and Nekrasov, 2019; Xu et al., 2019). As an alternative to permanent transgenic T-DNA-based expression systems, plant RNA viruses can be delivered via *Agrobacterium*-based systems to express genome-engineering reagents systemically in diverse plant species (MacFarlane, 1999; Macfarlane, 2010; Ratcliff et al., 2001). Plant RNA viruses, such as *Tobacco rattle virus* (TRV), have been efficiently used for virus-induced gene silencing (VIGS) or expressing the single guide RNA (sgRNA) of the CRISPR/Cas9 system for functional genomics or other biotechnological applications in plants (Ali et al., 2015a). TRV has bipartite, positive-sense, single-stranded RNA1 and RNA2 genomes (Macfarlane, 2010). Previously, we optimized and engineered the TRV RNA2 genome for effective systemic delivery of the sgRNA into *Nicotiana benthamiana* (Ali et al., 2015b, 2018).

Another possibility for delivering the CRISPR/Cas machinery would be to translocate Cas9 protein, rather than a construct encoding Cas9. Indeed, during *Agrobacterium*-mediated transformation, several virulence proteins are transported into the host cell through the T4SS and this transfer occurs independently of the T-DNA (Vergunst et al., 2000). A hydrophilic secretion signal with a net positive charge is responsible for the translocation of proteins through the T4SS (Vergunst et al., 2005). Several heterologous proteins, fused to this secretion signal, have been translocated through the T4SS, including the Cre recombinase and homing endonuclease I-SceI (Vergunst et al., 2000, 2005; Schrammeijer et al., 2003; Rolloos et al., 2015). For translocation, unfolding of these proteins is probably required, since green fluorescent protein (GFP), which is a small protein but with a rigid β-barrel structure, did not pass the T4SS channel (Vergunst et al., 2005). The size of the protein does not seem to limit transfer since the very large 1769 amino acid *Agrobacterium rhizogenes* GALLS protein fused to Cre recombinase can pass the T4SS (Hodges et al., 2006).

In this study we tested whether delivery of the Cas9 protein through the T4SS of *Agrobacterium* was possible and could lead to targeted mutagenesis in yeast and *N. benthamiana* plants if combined with a sgRNA. We tested whether sgRNAs could be co-delivered from *Agrobacterium*. Alternatively, sgRNAs were expressed in the recipient plant cells from a co-delivered T-DNA or viral vector. Our results revealed that genome editing is possible after translocation of the Cas9 protein, and this could therefore provide an alternative to the permanent and constitutive expression of Cas9 from an integrated T-DNA. As translocated Cas9 protein has a limited life span, this method could reduce the chance of off-target mutations.

## Materials and Methods

### Plasmid Construction

The HindIII/NotI restriction site-containing fragment with the *virF* promoter and the gene encoding a translation fusion between a nuclear localization signal (NLS), the Cre-recombinase, and the last 37 amino acids of the *Agrobacterium tumefaciens* virulence protein VirF (*virF* promoter-NLS::Cre::VirF37c) (Vergunst et al., 2000) from the pSDM3155 plasmid was inserted into the HindIII/NotI sites of the pUC18 plasmid to create the pSDM2131 plasmid. The Cre-recombinase fragment was removed by digestion of the pSDM2131 plasmid with SalI and BglII and was replaced by a small linker containing a BglII site (annealed oligos DS061 and DS062). The BamHI fragment encoding Cas9 from the pMJ920 plasmid (Addgene plasmid #42234) (Jinek et al., 2013) was inserted into the BglII site of pvirF-NLS::BglII Linker::VirF37c creating a translational fusion between a nuclear localization signal, Cas9, and the C-terminal 37 amino acids of VirF (NLS::Cas9::VirF37c) under the control of the *virF* promoter. The HindIII/NotI fragment with pvirF-NLS::Cas9::VirF37c was inserted into the HindII/NotI sites of the pBBR6 plasmid creating the pNCas9F plasmid (Figure S1).

For the expression of sgRNA under the bacterial promoters, the J23119 promoter (pJ) (Geng et al., 2019), the leader/promoter expressing the CRISPR array (pL) and the promoter expressing the tracr RNA (pT) (Jiang et al., 2013) linked to the sgRNA-PDS2 were custom synthesized and cloned into the NotI site of the pNCas9F plasmid. pVirF-NCas9 (without the VirF translocation signal) was constructed by removing the XbaI/SpeI fragment from NCas9F-pT-PDS-sgRNA containing the 37 aa VirF translocation signal and pT-PDS-sgRNA.

To create the sgRNA expression vectors, oligos DS196/DS197 were phosphorylated, annealed, and subsequently cloned into the BbsI site of the pEn-Chimera plasmid (Fauser et al., 2014). The correct clone was used as the entry vector for a Gateway LR reaction with the destination vector pDe-CAS9 (Fauser et al., 2014) resulting in the pDECas9-PDS1 plasmid, and the destination vector pMDC100 (Curtis and Grossniklaus, 2003) resulting in the sgRNA-PDS1 plasmid.

The RNA2- sgRNA-PDS2 construct was used for TRV-based expression of the sgRNA (Ali et al., 2015b). For the plasmid interference assay, the *PDS* target was amplified and cloned into the pENDER-D-Topo vector and sub-cloned into the binary vector pK2GW7 by the Gateway LR reaction.

### Co-cultivations of *Agrobacterium* With Yeast

Co-cultivations of *Agrobacterium* with the YPH499 yeast strain were carried out as previously described with the following minor modifications (Bundock et al., 2002). *Agrobacterium* was grown overnight at 29°C in LC with appropriate antibiotics. Induction of the *virF* promoter was performed at 28°C at OD_600_ = 0.25 for 6 h in induction medium containing 200 μM acetosyringone (Sigma-Aldrich Co.). The yeast strain with the p426-SNR52p-gRNA.CAN1.Y-SUP4t plasmid (Dicarlo et al., 2013) was grown overnight in MY minus the auxotrophic growth compound complemented by the plasmid and then diluted 10 times in YPD and propagated for 6 h. Yeast (10^7^ cells) and *Agrobacterium* (2^*^10^8^ cells) cells were mixed and were spotted on nitrocellulose filters on IM plates containing 200 μM acetosyringone (Sigma-Aldrich Co.) followed by an incubation at 21°C for 7 days.

### Mutation Analysis of the *CAN1* Locus

Yeast was recovered from the nitrocellulose filter and plated on SD medium containing L-canavanine sulfate (60 μg mL^−1^, Santa Cruz Biotechnology Inc.) to select for mutations of the *CAN1* locus and cefotaxime (200 μg mL^−1^, Formedium) to stop the growth of *Agrobacterium*. Total yeast cell numbers were determined by plating serial dilutions of yeast on YPD plates containing cefotaxime (200 μg mL^−1^, Formedium). The mutation frequency of the *CAN1* locus was determined by dividing the number of L-canavanine-resistant colonies by the total colony count based on the serial dilutions on YPD. Yeast was grown overnight in YPD at 30°C. Genomic DNA was isolated from 2 ml cultures using the YeaStar genomic DNA kit from Zymoclean (protocol 1). The *CAN1* locus was amplified from L-canavanine-resistant colonies by PCR with primers DS086 and DS088. The PCR fragments were cloned into the pJET2.1 vector (CloneJET PCR Cloning Kit, Thermo Fischer Inc.) before sequencing. The respective clones were subjected to Sanger sequencing (Macrogen Europe Inc.) and aligned to the wild-type sequence.

### Leaf Infiltration

*Nicotiana benthamiana* seeds were germinated and grown in controlled climate chambers at 24°C with a 16 h light /8 h dark photoperiod with 75% humidity for a period of 3 weeks before infiltration. *Agrobacterium* strains were grown overnight at 29°C with shaking at 180 RPM in a 10 ml culture of LC supplemented with appropriate antibiotics. The next day, *Agrobacterium* cells were resuspended in induction medium (Bundock et al., 1995) to an OD_600_ of 1.2 and were then kept at room temperature for 3 h without shaking. The *Agrobacterium* cells were then introduced into the two youngest leaves of 3-week-old *N. benthamiana* plants using needleless 1 ml syringes.

### TRV Infection and Sap Preparation

*N. benthamiana* plants were infiltrated with *Agrobacterium* harboring binary plasmids encoding TRV RNA 1 and engineered RNA2-sgRNA-PDS2. After 7 days, sap containing the RNA1 and engineered RNA2-sgRNA-PDS2 genomes was isolated in Potassium phosphate buffer (50 mM, pH 7.5) from non-infiltrated leaves and applied to *N. benthamiana* leaves using carborundum, that had been infiltrated 1 day earlier with *Agrobacterium* expressing NCas9F.

### DNA Isolation and Mutation Detection in Plants

Leaf discs were harvested 4–10 days after infiltration from which genomic DNA was isolated using the CTAB DNA extraction method (de Pater et al., 2009). Genomic DNA (500 ng) was pre-digested with DdeI (Thermo Scientific Inc) or NcoI (New England Biolabs) before the target sequence was amplified using primers DS192 and DS193 (490 bp PDS1 target) and M30 and M31 (283 bp PDS2 target; used for T7EI assay) or NBPDS3-gDNA-404bp-F and NBPDS3-gDNA-404bp-R (404 bp PDS2 target; used for NcoI digestion) (Table S1). For the loss of restriction site assay, the amplified products were digested with DdeI (Thermo Scientific Inc.) or NcoI (New England Biolabs). For Sanger sequencing, the restriction enzyme-resistant fragments were cloned into the pJET1.2 vector (Thermo Scientific Inc.). Individual clones were sent for Sanger sequencing (Macrogen Inc.). For the T7EI assay, 200 ng of the target flanking PCR product was reannealed (95-25°C with a 5°C per 2 min ramp down) and subjected to T7EI digestion (New England Biolabs). The DNA fragments were separated on 2% agarose gels.

### Amplicon Deep Sequencing

A total of 25 ng of DNA of 3–4 combined DNA samples was used for amplification with the primers SP669 and SP670 (Table S1) using Phusion High-Fidelity DNA Polymerase for 25 cycles. Products were purified by Ampure XP beads and used for 5 cycles of amplification with barcoded primers P5 and P7 (Tables S2, S3), after which the products were purified again by Ampure XP beads. Paired-end sequencing on an Illumina NovaSeq6000 was done using 1.1 nM DNA. Data analysis was performed using a custom sequencer-analyzer. In brief, paired-end reads were merged using PEAR (version 0.9.6) using standard settings and aligned reads were subsequently mapped to a reference sequence. Assembled reads with maximum base error-probability of 0.05 were kept for downstream analysis. Additionally, assembled reads needed to contain part of both primer sequences and only events that were different than the reference sequence were kept if the event started >5 bp from either primer annealing site. Based on the difference between the read and the reference an event was classified into: WT (no difference compared to the reference), Deletion, Delin (a deletion accompanied by an insertion), Insertion (piece of DNA inserted compared to the reference). Single nucleotide variations (substitutions) were not taken into account. Events were only included if they were seen at least twice. The data from multiple samples per transformation were combined (Table S2; **Figure 3D**).

## Results

### Translocation of the Cas9 Endonuclease

Agrobacteria can transform besides plants, also yeast and fungi (Bundock et al., 1995; Michielse et al., 2005) and apart from the T-DNA can translocate effector proteins into those species (Vergunst et al., 2000). Translocated effector proteins are characterized by a C-terminal arginine-rich translocation peptide (Vergunst et al., 2005). Here, we first used yeast (*Saccharomyces cerevisiae*) to test if the Cas9 protein could be translocated through the *Agrobacterium* T4SS. An expression plasmid was created encoding a fusion protein of Cas9 with a N-terminal nuclear localization signal and the C-terminal 37 amino acid translocation signal of the *Agrobacterium* virulence protein VirF. The expression of the fusion protein (NCas9F) was under the control of the acetosyringone-inducible *virF* promoter (Melchers et al., 1990) and 3′ flanking region (pVirF-NCas9F-tVirF) to ensure high expression and the presence of a functional T4SS. The *CAN1* gene was used as the target locus for induction of a DSB by the Cas9 endonuclease. This locus encodes a plasma membrane arginine transporter which mediates the uptake of arginine and its toxic analog L-canavanine into the cell. Mutation of *CAN1* leads to L-canavanine resistance. As a recipient, we used a yeast strain expressing a sgRNA targeting the *CAN1* locus driven from the strong *TEF1* promoter (Dicarlo et al., 2013). Therefore, in our experiments, translocation of NCas9F could be detected by an increased number of L-canavanine-resistant cells after co-cultivation with *Agrobacterium*.

**Figure 1:**
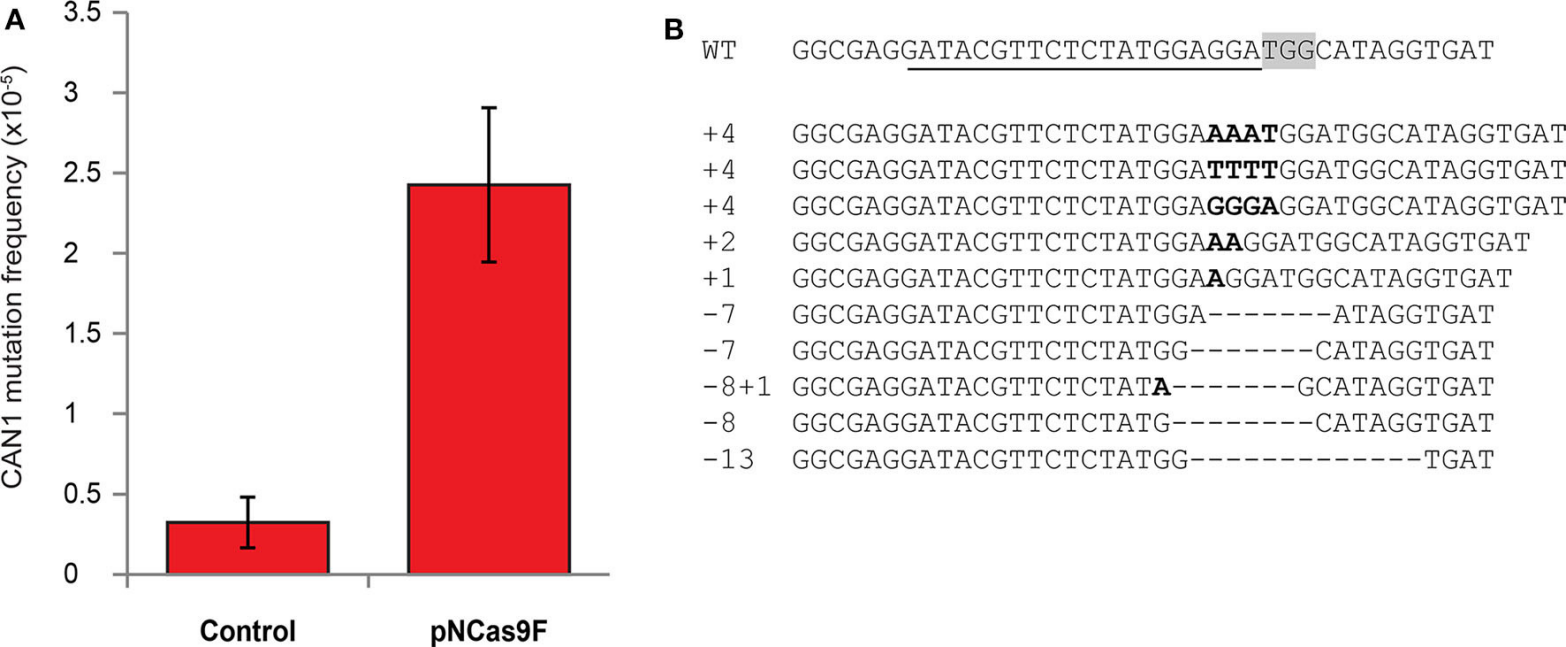
Mutagenesis via translocated Cas9 in yeast. **(A)** The mutation frequency of *CAN1* in yeast after co-cultivation with an *Agrobacterium* strain translocating NCas9F (pNCas9F) and an Agrobacterium strain lacking NCas9F (Control). Error bars indicate the SEM (*N* = 3). **(B)** Alignment of the wild-type *CAN1* target sequence with sequences obtained from L-canavanine-resistant yeast colonies after co-cultivation with an *Agrobacterium* strain translocating NCas9F. The PAM sequence is boxed and the recognition sequence of the sgRNA is underlined. Insertions are marked in bold and deletions are indicated by dashes.

The mutation frequency of *CAN1* in the recovered yeast cells was found to be ~2.5^*^10^−5^ after co-cultivation with *Agrobacterium* strain LBA1100 expressing NCas9F compared to ~0.2^*^10^−5^ after co-cultivation with the same *Agrobacterium* strain lacking the NCas9F expression plasmid (Figure 1A). To show that this ten-fold increase in the frequency of L-canavanine-resistant colonies was the consequence of the nuclease activity of the complex of the translocated NCas9F and sgRNA-CAN, we amplified and sequenced the *CAN1* locus of 16 L-canavanine-resistant colonies obtained after co-cultivation. This revealed that 15 of the 16 L-canavanine-resistant colonies had mutations directly upstream of the protospacer-adjacent motif (PAM) sequence (Figure 1B), whereas the *CAN1* locus of the 16th colony had a single base pair substitution 300 bp upstream of the PAM. The eight L-canavanine-resistant colonies obtained after co-cultivation with an *Agrobacterium* strain lacking NCas9F had mutations that were not located directly upstream of the PAM but were instead randomly distributed throughout the *CAN1* gene and therefore likely represented spontaneous mutations (Figure S2). These results showed that NCas9F was translocated through the T4SS and was able to induce DSBs in the presence of sgRNA.

### Targeted Mutagenesis in *Nicotiana benthamiana* Leaves After Cas9 Protein and sgRNA Translocation by *Agrobacterium*

**Figure 2:**
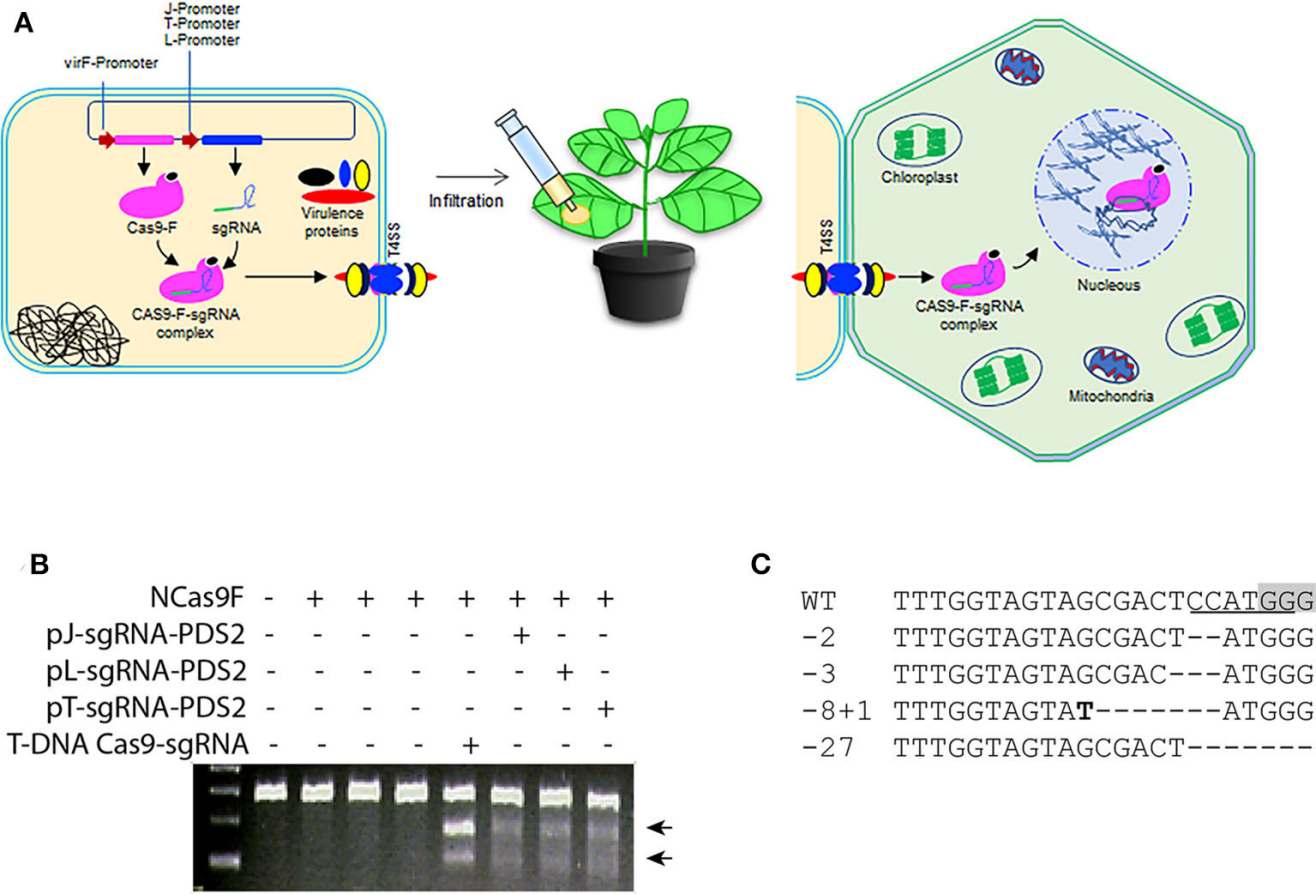
Translocation of NCas9F and sgRNA complex for targeted mutagenesis in *N. benthamiana*. **(A)** Schematic representation of the translocation of the NCas9F-sgRNA complex from *Agrobacterium* cells to plant cells. The sgRNA expression cassette under the control of the bacterial expression promoters were cloned individually into the plasmid expressing NCas9F to express the NCas9F-sgRNA complex in Agrobacteria. **(B)** Targeted mutagenesis via sgRNA-PDS2 expressed from the three bacterial promoters pJ, pT, and pL. Genomic DNA extracted from the infiltrated leaf discs was predigested with NcoI and used as template for amplification of the 283 bp *PDS* target fragment. The resulting PCR products were digested with T7EI endonuclease. T7EI-digested fragments (arrows) were observed from tissue co-transformed by NCas9F protein and sgRNA-PDS2. **(C)** Sequence alignment of the mutations after translocation of the NCas9F-sgRNA-PDS2 complex. The PAM sequence is gray and the NcoI site is underlined. Insertions are marked in bold and deletions are indicated by dashes.

The successful translocation of the functional NCas9F from *Agrobacterium* into yeast led us to test whether both components of the complex, NCas9F and the sgRNA, could be translocated via the T4SS of *Agrobacterium* to plant cells (Figure 2A). For these experiments, a target site (PDS2) in the genome of *Nicotiana benthaminiana* was chosen with an overlapping NcoI site. For expression of the sgRNA, we opted to use three different promoters to express the sgRNA-PDS2 in Agrobacteria: (1) the J23119 promoter (pJ) previously used for sgRNA expression in bacterial cells (Geng et al., 2019), (2) the promoter/leader expressing the CRISPR array (pL) or (3) the promoter expressing the tracr RNA (pT) of the of *Streptococcus pyogenes* CRISPR system (Jiang et al., 2013). These promoters were shown to be functional in *Streptococcus pneumonia* and *E.coli*. Since we did not know their activity in *Agrobacterium*, all three promoters were tested.

The pJ-sgRNA-PDS2, pL-sgRNA-PDS2, and pT-sgRNA-PDS2 cassettes were cloned individually into the plasmid expressing NCas9F. Plasmid interference assays were performed to confirm the production of an active NCas9F-sgRNA complex in *Agrobacterium* (Figure S2). To this end *Agrobacterium* strain AGL1 was transformed with the different NCas9F and sgRNA plasmids and the resulting strains were transformed with a plasmid with or without the PDS target and streaked on plates with the antibiotics for selection. Colonies were obtained when cells were selected for the presence of the plasmid lacking the PDS target site, but no colonies were obtained when cells were selected for the presence of the plasmid containing the PDS target site. This indicated that an active NCas9F/sgRNA nuclease complex was formed producing DSBs in the target leading to loss of the plasmid with the target site (Figure S3).

Subsequently, *Agrobacterium* strains harboring p(J, L, or T)-sgRNA-PDS2 and pVirF-NCas9F expression cassettes were infiltrated into *N. benthamiana* leaves and DNA samples were extracted from leaf discs. The genomic DNA was digested with NcoI to enrich for mutated targets where the NcoI, which overlapped the expected mutation site, was not intact. The digested DNA was used as a template to amplify a 283 bp fragment containing the target sequence by PCR. The purified PCR product was subjected to T7EI digestion to determine the presence of mutations at the target site. The appearance of T7EI-digested DNA fragments of the expected size demonstrated the formation of mutations at the target site (Figure 2B). To confirm the presence of these targeted modifications at the sequence level, the respective PCR products were cloned for Sanger sequencing. Sanger sequence reads of the targeted modification were aligned to the wild-type sequence (Figure 2C). Although the distribution of these mutations was similar to the mutations induced by T-DNA-expressed Cas9 and sgRNA, the frequencies of mutations were low, suggesting an inefficient translocation of the sgRNA-NCas9F complex via the T4SS.

### Targeted Mutagenesis in *Nicotiana benthamiana* Leaves With Translocated Cas9 Combined With sgRNA-Encoding T-DNA

**Figure 3:**
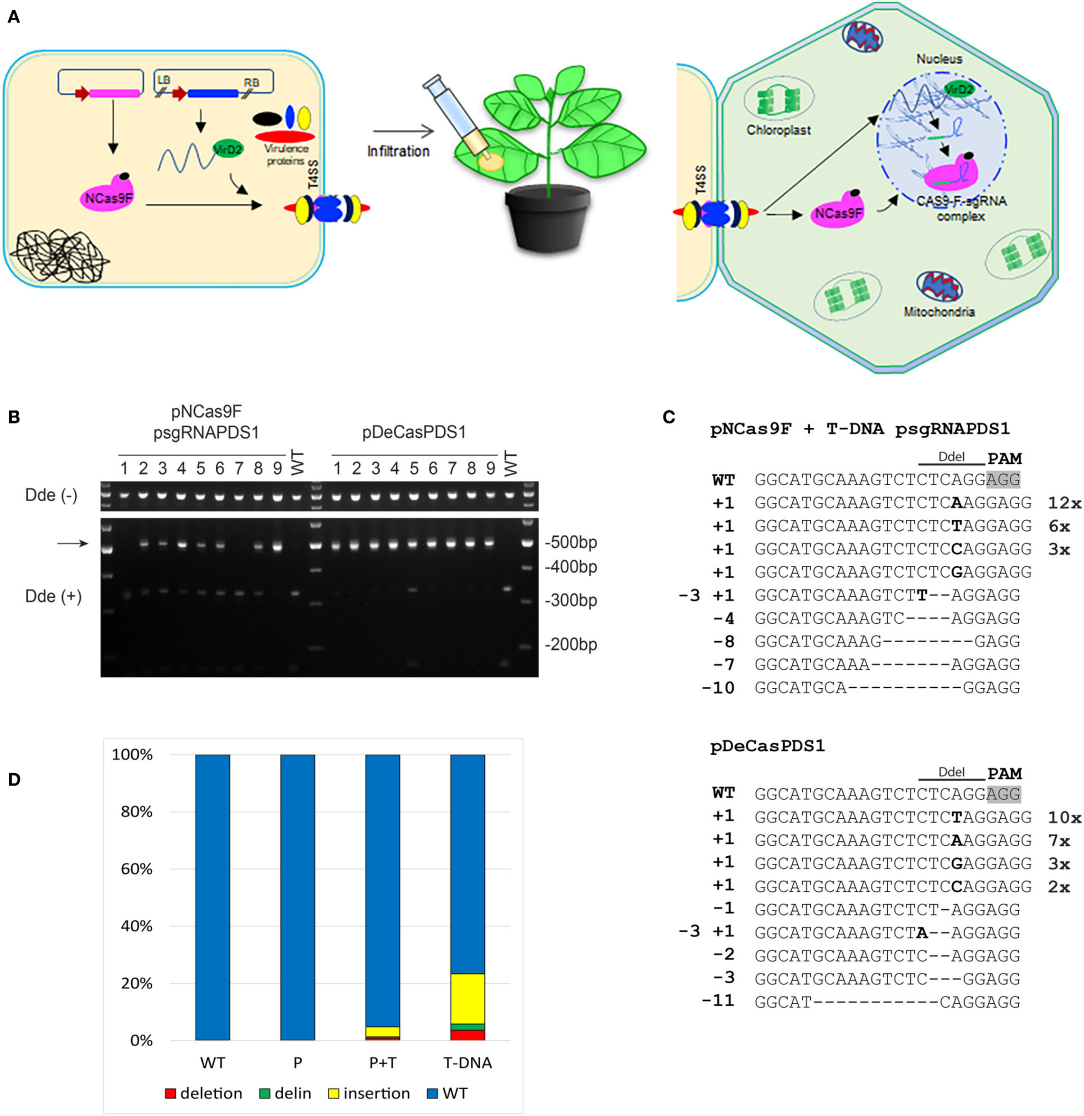
CRISPR/Cas9 endonuclease-induced mutagenesis in *N. benthamiana*. **(A)** Schematic presentation of NCas9F translocation together with T-DNA harboring the sgRNA-PDS1 expression cassette. **(B)** The *PDS1* target fragment was amplified from *N. benthamiana* genomic DNA, isolated from nine biological replicates (1–9), predigested with DdeI. The resulting 490 bp PCR products were digested with DdeI. DdeI-resistant 490 bp bands (arrow) were obtained from *N. benthamiana* tissue co-transformed with translocated NCas9F protein and a T-DNA encoding the sgRNA-PDS1 cassette or with T-DNA (pDeCasPDS1) encoding both Cas9 and sgRNA. **(C)** Sequence analysis of DdeI resistant PCR products after infiltration with NCas9F and T-DNA-sgRNA-PDS1 or pDECasPDS1. The DdeI site and PAM are indicated. Insertions are shown in bold, deletions with dashes. Length of insertions and deletions are shown at the left. Numbers of multiple clones with the same sequence are indicated at the right. **(D)** Deep sequencing analysis of the target sequence obtained from untreated tissue (WT) or tissue infiltrated with *Agrobacterium* transferring NCas9F protein alone (P) or combined with a T-DNA expressing the sgRNA (P+T), or infiltrated with *Agrobacterium* transferring a T-DNA expressing both Cas9 and sgRNA-PDS1 (T-DNA). The percentages of deletions (red), delins (green) and insertions (yellow) are indicated.

To increase the mutation frequency after translocation of NCas9F we combined protein translocation with translocation of T-DNA sgRNA-PDS1. For these experiments another target was chosen, with the sequence GG 5′of the PAM sequence since this may enhance the mutation frequency (Farboud and Meyer, 2015). *N. benthamiana* leaves were infiltrated with *Agrobacterium* strain AGL1 expressing the NCas9F protein and harboring a T-DNA expressing the sgRNA-PDS1 in plant cells (Figure 3A). As a control infiltrations were performed with AGL1 carrying a T-DNA that would express both Cas9 and the sgRNA-PDS1 in plant cells. Seven to ten days post infiltration, the tissue was harvested and the DNA was extracted. To easily detect mutations induced by the NCas9F nuclease, we used the loss-of-restriction-enzyme method (Voytas, 2013), as the target sequence of the sgRNA overlaps with a DdeI restriction site. To enrich for DNA molecules carrying mutations, the genomic DNA extracted from the infiltrated leaves was pre-digested with DdeI. A 490 bp fragment was then amplified by PCR with primers flanking the target sequence, and the resulting PCR products were again digested with DdeI. Restriction digestion-resistant bands were cloned and analyzed by sequencing (Figure 3B). As expected, translocation of a T-DNA encoding both Cas9 and the sgRNA resulted in restriction digestion-resistant PCR products in all of the nine infiltrated leaves. Translocation of the NCas9F protein together with a T-DNA encoding the sgRNA-PDS1 construct resulted in restriction digestion-resistant PCR products in seven out of nine infiltrated leaves (Figure 3B). Deletions ranging from 1 to 11 bp, single nucleotide insertions, and small deletions combined with insertions were found (Figure 3C). The sequences of the PCR products were very similar to those obtained after transformation by a T-DNA encoding both Cas9 and sgRNA, including deletions ranging from 1 to 9 bp, single nucleotide insertions, and combinations of deletions and insertions (Figure 3C). The results showed that NCas9F protein translocated through the T4SS into plant cells can form an active nuclease together with a sgRNA expressed from a translocated T-DNA.

Subsequently, we performed next generation sequencing (NGS) to compare the efficiency of targeted mutagenesis after NCas9F protein delivery with or without co-delivery of T-DNA encoding a sgRNA with that after transfer of a T-DNA encoding Cas9. Amplicons were generated by PCR from DNA isolated from 2 to 3 independently infiltrated leaves from 2 to 4 plants and these were combined for each of the two delivery methods. These PCR amplicons covering the protospacer and PAM sequences were sequenced on a NovaSeq6000 system and data analysis was done using a custom-made sequence analyzer tool. The mutations obtained with the positive control (Cas9 + sgRNA-PDS1 on a T-DNA) included insertions (1–72 bp), deletions (1–46 bp), and combinations of deletions and insertions (delins). The majority of the mutations were insertions. A mutation was present in around 23% of the reads (Figure 3D). Translocation of the NCas9F protein alone resulted in a low background of mutations as observed in untransformed tissue. However, translocation of the NCas9F protein together with T-DNA encoding sgRNA-PDS1 resulted in similar mutations (insertion of 1–27 bp, deletions of 1–49 bp and delins) in similar ratios as the positive control but at a 5-fold lower frequency (Figure 3D).

### Targeted Mutagenesis With Translocated NCas9F Protein and sgRNA Expressed by TRV

**Figure 4:**
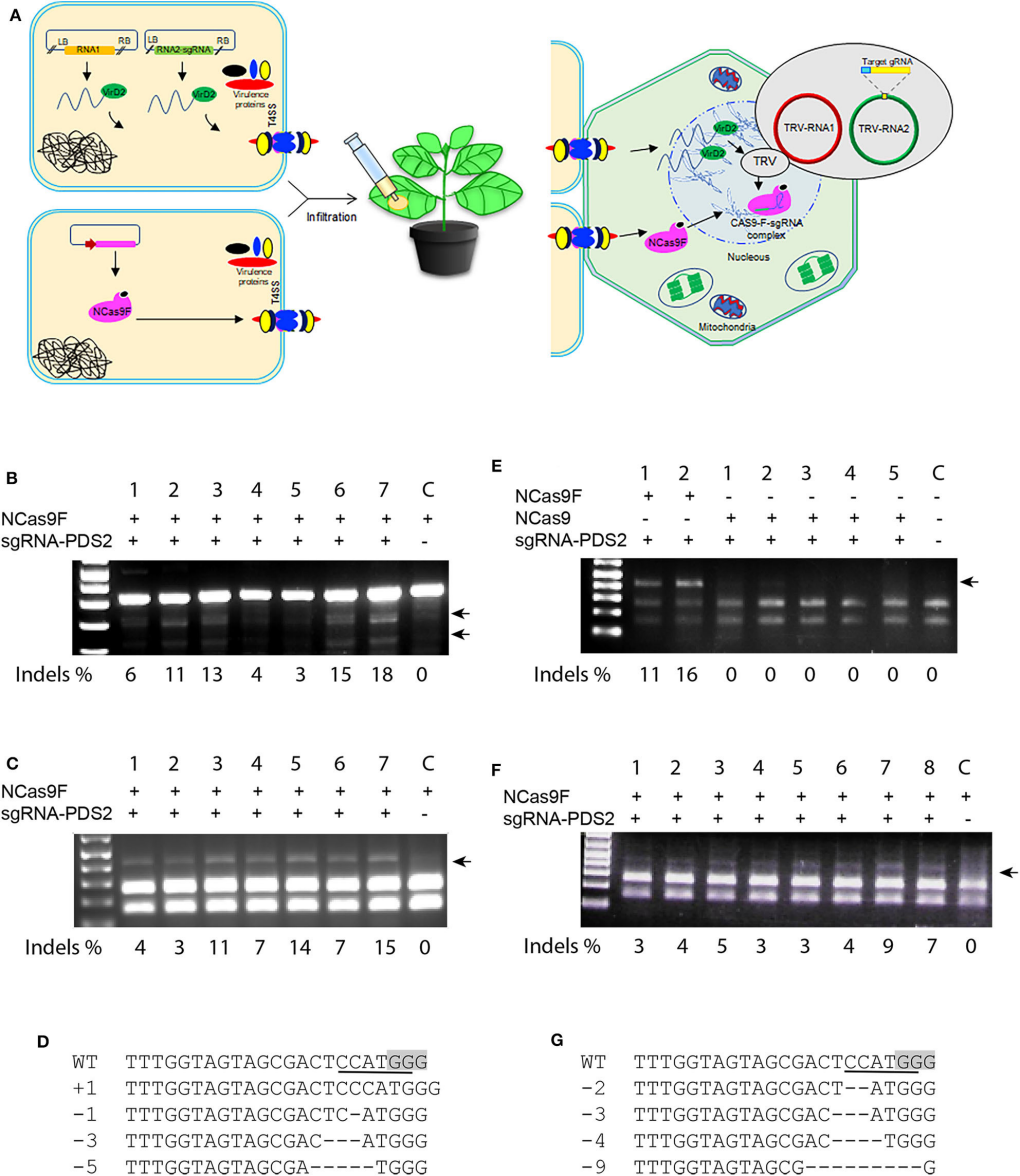
NCas9F translocation and TRV-based targeted mutagenesis in *N. benthamiana*. **(A)** Schematic presentation of the TRV-based sgRNA expression and NCas9F translocation. **(B)** The *PDS2* target fragment was amplified from *N. benthamiana* genomic DNA, isolated from seven biological replicates (1–7) and one control sample without sgRNA (C). The resulting 283 bp PCR products were digested with T7EI endonuclease. T7EI-digested fragments (arrows) were observed from tissue co-transformed with NCas9F protein and TRV encoding the sgRNA-PDS2 cassette. % indels is indicated. **(C)** Purified 404 bp PCR products were digested with NcoI to determine loss of the targeted restriction site. NcoI-resistant fragments (arrow) were observed from tissue co-transformed with NCas9F protein and TRV encoding the sgRNA-PDS2 cassette. % indels is indicated. **(D)** Alignment of the mutations after transformation with the NCas9F protein and TRV encoding the sgRNA-PDS cassette. **(E)** The *PDS2* target fragment was amplified from *N. benthamiana* genomic DNA, isolated from two biological NCas9F replicates (with VirF translocation signal), five biological NCas9 replicates (without translocation signal) and 1 untransformed control sample (C). The 404 bp PCR products were digested with NcoI. NcoI-resistant fragments (arrow) were observed from tissue co-transformed with NCas9F protein and TRV encoding the sgRNA-PDS2 cassette. % indels is indicated. **(F)** TRV was replicated in the wild-type *N. benthamiana* plants and the extracted sap containing viral RNA was applied to the *N. benthamiana* plants that had been infiltrated with Agrobacteria expressing NCas9F, 1 day earlier. The *PDS2* target fragment was amplified from the genomic DNA, isolated from eight biological replicates (1–8) and 1 control sample without sgRNA (C) and subjected to NcoI digestion. NcoI-resistant fragments (arrow) were observed from tissue co-transformed with NCas9F protein and enriched TRV encoding the sgRNA-PDS2 cassette. % indels is indicated. **(G)** Sequence analysis of mutations in NcoI-resistant PCR products shown in **(F)**. The PAM sequence is gray and the NcoI site is underlined. Deletions are indicated by dashes.

Translocation of the T-DNA harboring the sgRNA expression cassette and NCas9F protein may not always occur simultaneously into the same cell and thus can lower the targeted mutation frequencies in plants. To enhance the availability of the sgRNA in the leaf cells, we applied the TRV system to express the sgRNA. In contrast to T-DNA, the TRV system can spread to neighboring cells and systemically through the whole *N. benthamiana* plant (Ali et al., 2015a). *Agrobacterium* harboring TRV to express the sgRNA and *Agrobacterium* expressing NCas9F were simultaneously infiltrated into *N. benthamiana* leaves for targeted modification of the *PDS* locus (Figure 4A). For these experiments, the PDS2 target was used with an overlapping NcoI site. Genomic DNA was extracted and the target sequence was amplified directly by PCR. The 283 bp PCR fragments were subjected to T7EI digestion for detection of mutations. The presence of the expected T7EI digested DNA fragments were only detected in the samples expressing sgRNA-PDS2 from TRV that had received NCas9F after protein translocation from *Agrobacterium* (Figure 4B). Using semi-quantification using band intensities, targeted modification at the *PDS* locus was obtained in this case at a frequency from 3 to 18%. Although this cannot be directly compared to the NGS data, it indicated that the frequency was similar as that obtained after NCas9F protein translocation combined with T-DNA encoded sgRNA.

Subsequently, 404 bp PCR fragments containing the target sequence were subjected to NcoI digestion, since targeted modification could lead to loss of the NcoI recognition site. The appearance of NcoI-resistant DNA fragments confirmed the T7E1 assay results suggesting the formation of mutations at the target site (Figure 4C). Percentages of resistant bands were 3 to 15 %. The NcoI-resistant fragments were cloned into the pJET2.1 cloning vector and individual clones were analyzed by Sanger sequencing. The results confirmed the presence of mutations at the expected location (Figure 4D).

In order to exclude that the NCas9F protein was translocated by other means than the T4SS, agroinfiltration was performed using *Agrobacteria* expressing NCas9 without the C-terminal 37 amino acid translocation signal of the *Agrobacterium* virulence protein VirF mixed with *Agrobacterium* harboring TRV to express the sgRNA. *Agrobacteria* expressing NCas9F were used as positive control. The target site was amplified using genomic DNA as template and PCR products were digested by NcoI. The positive control samples showed 11 and 16% NcoI-resistant bands (Figure 4E). In contrast, no NcoI-resistant bands could be detected in the NCas9 samples. These results showed that the VirF translocation signal was required for gene editing and that NCas9F was translocated via the T4SS.

TRV multiplies in *N. benthamiana* plants and isolated sap containing the TRV viral RNA genomes is capable of re-infecting new plants. The viral RNA enrichment strategy can eliminate the need for T-DNA-based agroinfection of TRV. To confirm this, wild-type *N. benthamiana* plants were infiltrated with *Agrobacterium* harboring binary plasmids encoding TRV RNA1 and engineered RNA2-sgRNA-PDS2. Sap containing the RNA1 and engineered RNA2-sgRNA-PDS2 genomes was isolated and applied to *N. benthamiana* leaves already infiltrated 1 day earlier with *Agrobacterium* expressing NCas9F for targeted modification of the *PDS* locus. After 3–5 days, genomic DNA was extracted and the target site was amplified by PCR. The 404 bp PCR fragment was subjected to NcoI digestion to detect the loss of the targeted restriction site. The appearance of NcoI-resistant DNA fragments after translocation of NCas9F, only in the samples expressing sgRNA-PDS2, demonstrated the formation of mutations at the *PDS* locus (Figure 4F). Percentages of resistant bands were 3–9%. Sanger sequencing reads of the cloned NcoI-resistant DNA fragments confirmed the formation of targeted mutations at the *PDS2* target (Figure 4G).

## Discussion

In this study we demonstrated that the Cas9 protein of the type II bacterial CRISPR system can be translocated from *Agrobacterium* into yeast and plant via the T4SS, if provided with the translocation peptide of the *Agrobacterium* virulence protein VirF. This corroborates previous findings showing the translocation of heterologous proteins through this T4SS after fusion to the VirF translocation peptide (Vergunst et al., 2000, 2005; Schrammeijer et al., 2003; Hodges et al., 2006; Rolloos et al., 2015).

Mutations induced by the translocated NCas9F and T-DNA- or viral-expressed sgRNA were similar to mutations induced when Cas9 and sgRNA were both expressed from a T-DNA. However, the mutation frequencies were lower with translocated NCas9F protein and T-DNA-expressed sgRNA than with T-DNA-based expression of both Cas9 and sgRNA. The lower frequencies could be due to limiting levels of translocated NCas9F, the brief presence of NCas9F in the host after translocation, lower activity of the NCas9F protein compared to the Cas9 protein, or because transfer of T-DNA expressing sgRNA and the translocated NCas9F protein may not always occur simultaneously into the same cell. For targeted mutagenesis this approach would still be efficient in comparison to integration of T-DNA since no lines need to be selected with only one T-DNA copy and no progeny lines need to be isolated that have lost the T-DNA in the next generation. Furthermore, our strategies would be of great value in vegetatively propagated crops, where removal of integrated T-DNA is one of the main issues.

DNA free gene-editing has been successfully applied using other methods including delivery of *in vitro* produced transcripts or ribonucleoproteins (RNP) (Woo et al., 2015; Zhang et al., 2016; Chen et al., 2019). Such methods were also shown to reduce off-target mutations. However, for these approaches additional steps like isolation of protoplast or RNPs is required. For proteins translocation using *Agrobacterium* transformation no special skills are needed.

In our experiments using sgRNA-PDS1, small insertions, mainly consisting of 1 bp insertions, were detected 4–5 times more often than deletions. It has been reported that Cas9 might produce staggered ends with 5′overhangs, that will results in predictable 1 bp insertions after repair (Zuo and Liu, 2016; Shou et al., 2018). In previous experiments with *Arabidopsis thaliana* we did not observe this relatively high number of small insertions. Other reports on targeted mutagenesis in *N*. *benthamiana* with CRISPR/Cas9 genome editing using the loss-of-restriction enzyme method also did not detect high levels of small insertions (Nekrasov et al., 2013; Ali et al., 2015b; Lowder et al., 2015; Yin et al., 2015). High levels of small insertions were, however, reported in Arabidopsis after induction of DSBs with CRISPR/Cas9 using NGS (Fauser et al., 2014). This suggests that the outcome of repair of DSBs induced with CRISPR/Cas9 in plants is also dependent on the target sequence. Indeed, in mammalian cells it was shown that the sgRNA sequence determines the frequency of staggered ends produced by Cas9 activity (Gisler et al., 2019). This repair mechanisms might have been partly responsible for the observed A insertions in our experiments, but cannot explain the frequent T insertions. These single A and T insertions are consistent with the “A-rule” which states that polymerases are known to preferentially incorporate deoxyadenosine-monophosphate (dAMP) when template base coding is not available (Strauss, 2002).

When we tested whether both NCas9F and the sgRNA could be translocated from *Agrobacterium* into plant cells together, this resulted only in a very low level of targeted modifications. For translocation through the T4SS, most proteins need to become unfolded, since the size of the channel is too narrow (20–30 Å) (Li and Christie, 2018) to accommodate large or rigid proteins. Cas9 is a large multi-domain protein in complex with a sgRNA of about 10 nm (Shibata et al., 2017) and unfolding is required for transfer through the T4SS canal, probably resulting in a loss of the interaction with the sgRNA. Since the sgRNA contains no signal for transport through the T4SS, it probably will not enter the cell if the interaction with Cas9 is disrupted. Therefore, T-DNA-mediated or virus-mediated mechanisms for delivering the sgRNA proved much more effective.

To improve the transfer of the sgRNA into the majority of the cells, we used *Agrobacterium* for delivery of a TRV system for sgRNA expression. After initial expression from the T-DNA, TRV can spread sgRNA into the leaf cells more efficiently than a standard T-DNA, as TRV -once formed- not only can replicate, but also can move from cell to cell through plasmodesmata (Ali et al., 2015b). One of the other advantages of the TRV system is that the RNA genomes (RNA1 and RNA2-sgRNA) become enriched in plants by replication, and the sap extracted from such plants containing RNA1 and RNA2-sgRNA can be used to infect new plants. When such sap is applied to plants infected by an *Agrobacterium* strain that can translocate NCas9F, this can lead to targeted mutations. Any mutant recovered from this system will be completely T-DNA free, which is highly desired in plant biotechnology and crop breeding. In addition, viral RNA can be lost in next generations or in regenerated plants from leaf tissue, which would result in plants mutated at the target locus but without any transgenes.

In summary, our experimental data confirmed the efficient delivery of the NCas9F protein into yeast and plant cells via the T4SS of *Agrobacterium*. This can be used as an alternative to T-DNA-based transgenic systems for generation of non-transgenic targeted modifications in the plant genome.

## Data Availability Statement

The datasets presented in this study can be found in online repositories. The names of the repository/repositories and accession number(s) can be found below: https://www.ncbi.nlm.nih.gov/, Bioproject ID PRJNA622227; https://www.ncbi.nlm.nih.gov/, Bioproject ID PRJNA634985.

## Author Contributions

PH, MM, and SP conceived and supervised the research. DS, ZA, PH, MM, and SP wrote the article. DS, CW, ZA, FA, and SP performed the experiments and analyzed the results. All authors contributed to the article and approved the submitted version.

## Conflict of Interest

PH, MM, and SP are authors on patents pertaining to plant genome engineering tools and methods. The remaining authors declare that the research was conducted in the absence of any commercial or financial relationships that could be construed as a potential conflict of interest.
