# Supplementary material for: CRISPR/Cas9 Mutagenesis by Translocation of Cas9 Protein Into Plant Cells via the Agrobacterium Type IV Secretion System
Source: Front Genome Ed. 2020 Jul 17;2:6. doi: 10.3389/fgeed.2020.00006 (PMC8525350; doi:10.3389/fgeed.2020.00006)
Supplement: Supplementary file 1 [file Data_Sheet_1.pdf]

SUPPLEMENTARY MATERIAL

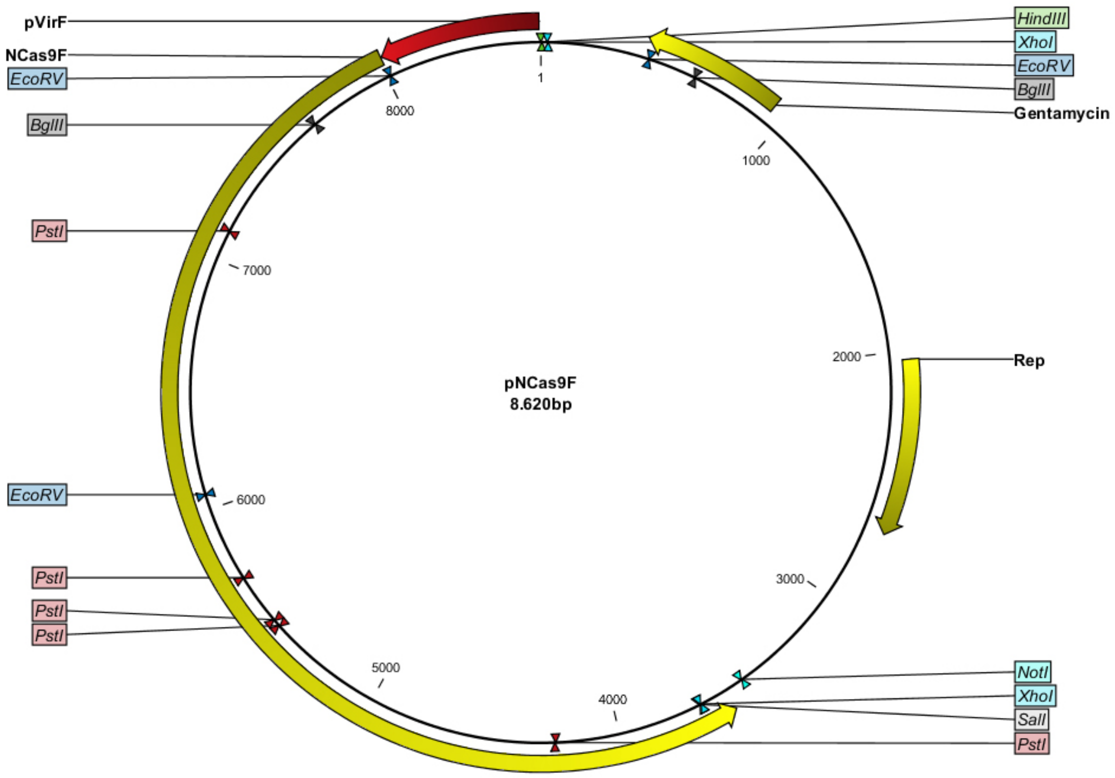

Figure S1 Plasmid pNCas9F for expression of Cas9 in *Agrobacterium* and translocation via the T4SS. An NLS has been fused to the N terminus of Cas9 and 37 C terminal amino acids of VirF has been fused to the C terminus of Cas9. The fusion protein is under control of the *virF* promoter and *virF* 3'UTR.

Figure S2 Sequences of the CAN1 locus from eight yeast colonies obtained after a co-cultivation with an Agrobacterium strain lacking NCas9F. Substitutions are marked in red, insertion are marked in cyan, deletions are underlined, the protospacer sequence is marked in light green and the protospacer adjacent motive (PAM) is marked in dark green.

```
>1 G to T substitution at position 509 and T to C substitution at position 1346
ATGACAAATTCAAAAGAAGACGCCGACATAGAGGAGAAGCATATGTACAATGAGCCGGTCACAACCCTCTTTCACGACGTTGAAGCTTCACAAA
CACACCACAGACGTGGGTCAATACCATTGAAAGATGAGAAAAGTAAAGAATTGTATCCATTGCGCTCTTTCCCGACGAGAGTAAATGGCGAGGA
TACGTTCTCTATGGAGGATGGCATAGGTGATGAAGATGAAGGAGAAGTACAGAACGCTGAAGTGAAGAGAGAGCTTAAGCAAAGACATATTGGT
ATGATTGCCCTTGGTGGTACTATTGGTACAGGTCTTTTCATTGGTTTATCCACACCTCTGACCAACGCCGGCCCAGTGGGCGCTCTTATATCAT
ATTTATTTATGGGTTCTTTGGCATATTCTGTCACGCAGTCCTTGGGTGAAATGGCTACATTCATCCCTGTTACATCCTCTTTCACAGTTTTCTC
ACAAAGATTCCTTTCTCCAGCATTTGGTGCGGCCAATGATTACATGTATTGGTTTTCTTGGGCAATCACTTTTGCCCTGGAACTTAGTGTAGTT
GGCCAAGTCATTCAATTTTGGACGTACAAAGTTCCACTGGCGGCATGGATTAGTATTTTTTGGGTAATTATCACAATAATGAACTTGTTCCCTG
TCAAATATTACGGTGAATTCGAGTTCTGGGTCGCTTCCATCAAAGTTTTAGCCATTATCGGGTTTCTAATATACTGTTTTTGTATGGTTTGTGG
TGCTGGGGTTACCGGCCCAGTTGGATTCCGTTATTGGAGAAACCCAGGTGCCTGGGGTCCAGGTATAATATCTAAGGATAAAAACGAAGGGAGG
TTCTTAGGTTGGGTTTCCTCTTTGATTAACGCTGCCTTCACATTTCAAGGTACTGAACTAGTTGGTATCACTGCTGGTGAAGCTGCAAACCCCA
GAAAATCCGTTCCAAGAGCCATCAAAAAAGTTGTTTTCCGTATCTTAACCTTCTACATTGGCTCTCTATTATTCATTGGACTTTTAGTTCCATA
CAATGACCCTAAACTAACACAATCTACTTCCTACGTTTCTACTTCTCCCTTTATTATTGCTATTGAGAACTCTGGTACAAAGGTTTTGCCACAT
ATCTTCAACGCTGTTATCTTAACAACCATTATTTCTGCCGCAAATTCAAATATTTACGTTGGTTCCCGTATTTTATTTGGTCTATCAAAGAACA
AGTTGGCTCCTAAATTCCTGTCAAGGACCACCAAAGGTGGTGTTCCATACATTGCAGTTTTCGTTACTGCTGCATTTGGCGCTTTGGCTTACAT
GGAGACATCTACTGGTGGTGACAAAGTTTCCGAATGGCTATTAAATATCACTGGTGTTGCAGGCTTTTTTGCATGGTTATTTATCTCAATCTCG
CACATCAGATTTATGCAAGCTTTGAAATACCGTGGCATCTCTCGTGACGAGTTACCATTTAAAGCTAAATTAATGCCCGGCTTGGCTTATTATG
CGGCCACATTTATGACGATCATTATCATTATTCAAGGTTTCACGGCTTTTGCACCAAAATTCAATGGTGTTAGCTTTGCTGCCGCCTATATCTC
TATTTTCCTGTTCTTAGCTGTTTGGATCTTATTTCAATGCATATTCAGATGCAGATTTATTTGGAAGATTGGAGATGTCGACATCGATTCCGAT
AGAAGAGACATTGAGGCAATTGTATGGGAAGATCATGAACCAAAGACTTTTTGGGACAAATTTTGGAATGTTGTAGCATAG
```

```
>2 T to C substitution at position 667
ATGACAAATTCAAAAGAAGACGCCGACATAGAGGAGAAGCATATGTACAATGAGCCGGTCACAACCCTCTTTCACGACGTTGAAGCTTCACAAA
CACACCACAGACGTGGGTCAATACCATTGAAAGATGAGAAAAGTAAAGAATTGTATCCATTGCGCTCTTTCCCGACGAGAGTAAATGGCGAGGA
TACGTTCTCTATGGAGGATGGCATAGGTGATGAAGATGAAGGAGAAGTACAGAACGCTGAAGTGAAGAGAGAGCTTAAGCAAAGACATATTGGT
ATGATTGCCCTTGGTGGTACTATTGGTACAGGTCTTTTCATTGGTTTATCCACACCTCTGACCAACGCCGGCCCAGTGGGCGCTCTTATATCAT
ATTTATTTATGGGTTCTTTGGCATATTCTGTCACGCAGTCCTTGGGTGAAATGGCTACATTCATCCCTGTTACATCCTCTTTCACAGTTTTCTC
ACAAAGATTCCTTTCTCCAGCATTTGGTGCGGCCAATGGTTACATGTATTGGTTTTCTTGGGCAATCACTTTTGCCCTGGAACTTAGTGTAGTT
GGCCAAGTCATTCAATTTTGGACGTACAAAGTTCCACTGGCGGCATGGATTAGTATTTTTTGGGTAATTATCACAATAATGAACTTGTTCCCTG
TCAAATATCACGGTGAATTCGAGTTCTGGGTCGCTTCCATCAAAGTTTTAGCCATTATCGGGTTTCTAATATACTGTTTTTGTATGGTTTGTGG
TGCTGGGGTTACCGGCCCAGTTGGATTCCGTTATTGGAGAAACCCAGGTGCCTGGGGTCCAGGTATAATATCTAAGGATAAAAACGAAGGGAGG
TTCTTAGGTTGGGTTTCCTCTTTGATTAACGCTGCCTTCACATTTCAAGGTACTGAACTAGTTGGTATCACTGCTGGTGAAGCTGCAAACCCCA
GAAAATCCGTTCCAAGAGCCATCAAAAAAGTTGTTTTCCGTATCTTAACCTTCTACATTGGCTCTCTATTATTCATTGGACTTTTAGTTCCATA
CAATGACCCTAAACTAACACAATCTACTTCCTACGTTTCTACTTCTCCCTTTATTATTGCTATTGAGAACTCTGGTACAAAGGTTTTGCCACAT
ATCTTCAACGCTGTTATCTTAACAACCATTATTTCTGCCGCAAATTCAAATATTTACGTTGGTTCCCGTATTTTATTTGGTCTATCAAAGAACA
AGTTGGCTCCTAAATTCCTGTCAAGGACCACCAAAGGTGGTGTTCCATACATTGCAGTTTTCGTTACTGCTGCATTTGGCGCTTTGGCTTACAT
GGAGACATCTACTGGTGGTGACAAAGTTTTCGAATGGCTATTAAATATCACTGGTGTTGCAGGCTTTTTTGCATGGTTATTTATCTCAATCTCG
CACATCAGATTTATGCAAGCTTTGAAATACCGTGGCATCTCTCGTGACGAGTTACCATTTAAAGCTAAATTAATGCCCGGCTTGGCTTATTATG
CGGCCACATTTATGACGATCATTATCATTATTCAAGGTTTCACGGCTTTTGCACCAAAATTCAATGGTGTTAGCTTTGCTGCCGCCTATATCTC
TATTTTCCTGTTCTTAGCTGTTTGGATCTTATTTCAATGCATATTCAGATGCAGATTTATTTGGAAGATTGGAGATGTCGACATCGATTCCGAT
AGAAGAGACATTGAGGCAATTGTATGGGAAGATCATGAACCAAAGACTTTTTGGGACAAATTTTGGAATGTTGTAGCATAG
```

```
>3 Deletion of A at position 1576 and insertion of TTTTT at position 1576
ATGACAAATTCAAAAGAAGACGCCGACATAGAGGAGAAGCATATGTACAATGAGCCGGTCACAACCCTCTTTCACGACGTTGAAGCTTCACAAA
CACACCACAGACGTGGGTCAATACCATTGAAAGATGAGAAAAGTAAAGAATTGTATCCATTGCGCTCTTTCCCGACGAGAGTAAATGGCGAGGA
TACGTTCTCTATGGAGGATGGCATAGGTGATGAAGATGAAGGAGAAGTACAGAACGCTGAAGTGAAGAGAGAGCTTAAGCAAAGACATATTGGT
ATGATTGCCCTTGGTGGTACTATTGGTACAGGTCTTTTCATTGGTTTATCCACACCTCTGACCAACGCCGGCCCAGTGGGCGCTCTTATATCAT
ATTTATTTATGGGTTCTTTGGCATATTCTGTCACGCAGTCCTTGGGTGAAATGGCTACATTCATCCCTGTTACATCCTCTTTCACAGTTTTCTC
ACAAAGATTCCTTTCTCCAGCATTTGGTGCGGCCAATGGTTACATGTATTGGTTTTCTTGGGCAATCACTTTTGCCCTGGAACTTAGTGTAGTT
GGCCAAGTCATTCAATTTTGGACGTACAAAGTTCCACTGGCGGCATGGATTAGTATTTTTTGGGTAATTATCACAATAATGAACTTGTTCCCTG
TCAAATATTACGGTGAATTCGAGTTCTGGGTCGCTTCCATCAAAGTTTTAGCCATTATCGGGTTTCTAATATACTGTTTTTGTATGGTTTGTGG
TGCTGGGGTTACCGGCCCAGTTGGATTCCGTTATTGGAGAAACCCAGGTGCCTGGGGTCCAGGTATAATATCTAAGGATAAAAACGAAGGGAGG
TTCTTAGGTTGGGTTTCCTCTTTGATTAACGCTGCCTTCACATTTCAAGGTACTGAACTAGTTGGTATCACTGCTGGTGAAGCTGCAAACCCCA
GAAAATCCGTTCCAAGAGCCATCAAAAAAGTTGTTTTCCGTATCTTAACCTTCTACATTGGCTCTCTATTATTCATTGGACTTTTAGTTCCATA
CAATGACCCTAAACTAACACAATCTACTTCCTACGTTTCTACTTCTCCCTTTATTATTGCTATTGAGAACTCTGGTACAAAGGTTTTGCCACAT
ATCTTCAACGCTGTTATCTTAACAACCATTATTTCTGCCGCAAATTCAAATATTTACGTTGGTTCCCGTATTTTATTTGGTCTATCAAAGAACA
AGTTGGCTCCTAAATTCCTGTCAAGGACCACCAAAGGTGGTGTTCCATACATTGCAGTTTTCGTTACTGCTGCATTTGGCGCTTTGGCTTACAT
GGAGACATCTACTGGTGGTGACAAAGTTTTCGAATGGCTATTAAATATCACTGGTGTTGCAGGCTTTTTTGCATGGTTATTTATCTCAATCTCG
CACATCAGATTTATGCAAGCTTTGAAATACCGTGGCATCTCTCGTGACGAGTTACCATTTAAAGCTAAATTAATGCCCGGCTTGGCTTATTATG
CGGCCACATTTATGACGATCATTATCATTATTCAAGGTTTCACGGCTTTTGCACCAAAATTCAATGGTGTT_TTTTTGCTTTGCTGCCGCCTAT
ATCTCTATTTTCCTGTTCTTAGCTGTTTGGATCTTATTTCAATGCATATTCAGATGCAGATTTATTTGGAAGATTGGAGATGTCGACATCGATT
CCGATAGAAGAGACATTGAGGCAATTGTATGGGAAGATCATGAACCAAAGACTTTTTGGGACAAATTTTGGAATGTTGTAGCATAG
```

```
>4 Insertion of GTGTTG at position 1376
ATGACAAATTCAAAAGAAGACGCCGACATAGAGGAGAAGCATATGTACAATGAGCCGGTCACAACCCTCTTTCACGACGTTGAAGCTTCACAAA
CACACCACAGACGTGGGTCAATACCATTGAAAGATGAGAAAAGTAAAGAATTGTATCCATTGCGCTCTTTCCCGACGAGAGTAAATGGCGAGGA
TACGTTCTCTATGGAGGATGGCATAGGTGATGAAGATGAAGGAGAAGTACAGAACGCTGAAGTGAAGAGAGAGCTTAAGCAAAGACATATTGGT
ATGATTGCCCTTGGTGGTACTATTGGTACAGGTCTTTTCATTGGTTTATCCACACCTCTGACCAACGCCGGCCCAGTGGGCGCTCTTATATCAT
ATTTATTTATGGGTTCTTTGGCATATTCTGTCACGCAGTCCTTGGGTGAAATGGCTACATTCATCCCTGTTACATCCTCTTTCACAGTTTTCTC
ACAAAGATTCCTTTCTCCAGCATTTGGTGCGGCCAATGGTTACATGTATTGGTTTTCTTGGGCAATCACTTTTGCCCTGGAACTTAGTGTAGTT
```

GGCCAAGTCATTCAATTTTGGACGTACAAAGTTCCACTGGCGGCATGGATTAGTATTTTTTGGGTAATTATCACAATAATGAACTTGTTCCCTG
TCAAATATTACGGTGAATTCGAGTTCTGGGTCGCTTCCATCAAAGTTTTAGCCATTATCGGGTTTCTAATATACTGTTTTTGTATGGTTTGTGG
TGCTGGGGTTACCGGCCCAGTTGGATTCCGTTATTGGAGAAACCCAGGTGCCTGGGGTCCAGGTATAATATCTAAGGATAAAAACGAAGGGAGG
TTCTTAGGTTGGGTTTCCTCTTTGATTAACGCTGCCTTCACATTTCAAGGTACTGAACTAGTTGGTATCACTGCTGGTGAAGCTGCAAACCCCA
GAAAATCCGTTCCAAGAGCCATCAAAAAAGTTGTTTTCCGTATCTTAACCTTCTACATTGGCTCTCTATTATTCATTGGACTTTTAGTTCCATA
CAATGACCCTAAACTAACACAATCTACTTCCTACGTTTCTACTTCTCCCTTTATTATTGCTATTGAGAACTCTGGTACAAAGGTTTTGCCACAT
ATCTTCAACGCTGTTATCTTAACAACCATTATTTCTGCCGCAAATTCAAATATTTACGTTGGTTCCCGTATTTTATTTGGTCTATCAAAGAACA
AGTTGGCTCCTAAATTCCTGTCAAGGACCACCAAAGGTGGTGTTCCATACATTGCAGTTTTCGTTACTGCTGCATTTGGCGCTTTGGCTTACAT
GGAGACATCTACTGGTGGTGACAAAGTTTTCGAATGGCTATTAAATATCACTGGTGTTGGTGTTGCAGGCTTTTTTGCATGGTTATTTATCTCA
ATCTCGCACATCAGATTTATGCAAGCTTTGAAATACCGTGGCATCTCTCGTGACGAGTTACCATTTAAAGCTAAATTAATGCCCGGCTTGGCTT
ATTATGCGGCCACATTTATGACGATCATTATCATTATTCAAGGTTTCACGGCTTTTGCACCAAAATTCAATGGTGTTAGCTTTGCTGCCGCCTA
TATCTCTATTTTCCTGTTCTTAGCTGTTTGGATCTTATTTCAATGCATATTCAGATGCAGATTTATTTGGAAGATTGGAGATGTCGACATCGAT
TCCGATAGAAGAGACATTGAGGCAATTGTATGGGAAGATCATGAACCAAAGACTTTTTGGGACAAATTTTGGAATGTTGTAGCATAG

>5 Insertion of T at position 626
ATGACAAATTCAAAAGAAGACGCCGACATAGAGGAGAAGCATATGTACAATGAGCCGGTCACAACCCTCTTTCACGACGTTGAAGCTTCACAAA
CACACCACAGACGTGGGTCAATACCATTGAAAGATGAGAAAAGTAAAGAATTGTATCCATTGCGCTCTTTCCCGACGAGAGTAAATGGCGAGGA
TACGTTCTCTATGGAGGATGGCATAGGTGATGAAGATGAAGGAGAAGTACAGAACGCTGAAGTGAAGAGAGAGCTTAAGCAAAGACATATTGGT
ATGATTGCCCTTGGTGGTACTATTGGTACAGGTCTTTTCATTGGTTTATCCACACCTCTGACCAACGCCGGCCCAGTGGGCGCTCTTATATCAT
ATTTATTTATGGGTTCTTTGGCATATTCTGTCACGCAGTCCTTGGGTGAAATGGCTACATTCATCCCTGTTACATCCTCTTTCACAGTTTTCTC
ACAAAGATTCCTTTCTCCAGCATTTGGTGCGGCCAATGGTTACATGTATTGGTTTTCTTGGGCAATCACTTTTGCCCTGGAACTTAGTGTAGTT
GGCCAAGTCATTCAATTTTGGACGTACAAAGTTCCACTGGCGGCATGGATTAGTATTTTTTTGGGTAATTATCACAATAATGAACTTGTTCCCT
GTCAAATATTACGGTGAATTCGAGTTCTGGGTCGCTTCCATCAAAGTTTTAGCCATTATCGGGTTTCTAATATACTGTTTTTGTATGGTTTGTG
GTGCTGGGGTTACCGGCCCAGTTGGATTCCGTTATTGGAGAAACCCAGGTGCCTGGGGTCCAGGTATAATATCTAAGGATAAAAACGAAGGGAG
GTTCTTAGGTTGGGTTTCCTCTTTGATTAACGCTGCCTTCACATTTCAAGGTACTGAACTAGTTGGTATCACTGCTGGTGAAGCTGCAAACCCC
AGAAAATCCGTTCCAAGAGCCATCAAAAAAGTTGTTTTCCGTATCTTAACCTTCTACATTGGCTCTCTATTATTCATTGGACTTTTAGTTCCAT
ACAATGACCCTAAACTAACACAATCTACTTCCTACGTTTCTACTTCTCCCTTTATTATTGCTATTGAGAACTCTGGTACAAAGGTTTTGCCACA
TATCTTCAACGCTGTTATCTTAACAACCATTATTTCTGCCGCAAATTCAAATATTTACGTTGGTTCCCGTATTTTATTTGGTCTATCAAAGAAC
AAGTTGGCTCCTAAATTCCTGTCAAGGACCACCAAAGGTGGTGTTCCATACATTGCAGTTTTCGTTACTGCTGCATTTGGCGCTTTGGCTTACA
TGGAGACATCTACTGGTGGTGACAAAGTTTTCGAATGGCTATTAAATATCACTGGTGTTGCAGGCTTTTTTGCATGGTTATTTATCTCAATCTC
GCACATCAGATTTATGCAAGCTTTGAAATACCGTGGCATCTCTCGTGACGAGTTACCATTTAAAGCTAAATTAATGCCCGGCTTGGCTTATTAT
GCGGCCACATTTATGACGATCATTATCATTATTCAAGGTTTCACGGCTTTTGCACCAAAATTCAATGGTGTTAGCTTTGCTGCCGCCTATATCT
CTATTTTCCTGTTCTTAGCTGTTTGGATCTTATTTCAATGCATATTCAGATGCAGATTTATTTGGAAGATTGGAGATGTCGACATCGATTCCGA
TAGAAGAGACATTGAGGCAATTGTATGGGAAGATCATGAACCAAAGACTTTTTGGGACAAATTTTGGAATGTTGTAGCATAG

>6 G to T substitution at position 272
ATGACAAATTCAAAAGAAGACGCCGACATAGAGGAGAAGCATATGTACAATGAGCCGGTCACAACCCTCTTTCACGACGTTGAAGCTTCACAAA
CACACCACAGACGTGGGTCAATACCATTGAAAGATGAGAAAAGTAAAGAATTGTATCCATTGCGCTCTTTCCCGACGAGAGTAAATGGCGAGGA
TACGTTCTCTATGGAGGATGGCATAGGTGATGAAGATGAAGGAGAAGTACAGAACGCTGAAGTGAAGAGAGAGCTTAAGCAAATACATATTGGT
ATGATTGCCCTTGGTGGTACTATTGGTACAGGTCTTTTCATTGGTTTATCCACACCTCTGACCAACGCCGGCCCAGTGGGCGCTCTTATATCAT
ATTTATTTATGGGTTCTTTGGCATATTCTGTCACGCAGTCCTTGGGTGAAATGGCTACATTCATCCCTGTTACATCCTCTTTCACAGTTTTCTC
ACAAAGATTCCTTTCTCCAGCATTTGGTGCGGCCAATGGTTACATGTATTGGTTTTCTTGGGCAATCACTTTTGCCCTGGAACTTAGTGTAGTT
GGCCAAGTCATTCAATTTTGGACGTACAAAGTTCCACTGGCGGCATGGATTAGTATTTTTTGGGTAATTATCACAATAATGAACTTGTTCCCTG
TCAAATATTACGGTGAATTCGAGTTCTGGGTCGCTTCCATCAAAGTTTTAGCCATTATCGGGTTTCTAATATACTGTTTTTGTATGGTTTGTGG
TGCTGGGGTTACCGGCCCAGTTGGATTCCGTTATTGGAGAAACCCAGGTGCCTGGGGTCCAGGTATAATATCTAAGGATAAAAACGAAGGGAGG
TTCTTAGGTTGGGTTTCCTCTTTGATTAACGCTGCCTTCACATTTCAAGGTACTGAACTAGTTGGTATCACTGCTGGTGAAGCTGCAAACCCCA
GAAAATCCGTTCCAAGAGCCATCAAAAAAGTTGTTTTCCGTATCTTAACCTTCTACATTGGCTCTCTATTATTCATTGGACTTTTAGTTCCATA
CAATGACCCTAAACTAACACAATCTACTTCCTACGTTTCTACTTCTCCCTTTATTATTGCTATTGAGAACTCTGGTACAAAGGTTTTGCCACAT
ATCTTCAACGCTGTTATCTTAACAACCATTATTTCTGCCGCAAATTCAAATATTTACGTTGGTTCCCGTATTTTATTTGGTCTATCAAAGAACA
AGTTGGCTCCTAAATTCCTGTCAAGGACCACCAAAGGTGGTGTTCCATACATTGCAGTTTTCGTTACTGCTGCATTTGGCGCTTTGGCTTACAT
GGAGACATCTACTGGTGGTGACAAAGTTTTCGAATGGCTATTAAATATCACTGGTGTTGCAGGCTTTTTTGCATGGTTATTTATCTCAATCTCG
CACATCAGATTTATGCAAGCTTTGAAATACCGTGGCATCTCTCGTGACGAGTTACCATTTAAAGCTAAATTAATGCCCGGCTTGGCTTATTATG
CGGCCACATTTATGACGATCATTATCATTATTCAAGGTTTCACGGCTTTTGCACCAAAATTCAATGGTGTTAGCTTTGCTGCCGCCTATATCTC
TATTTTCCTGTTCTTAGCTGTTTGGATCTTATTTCAATGCATATTCAGATGCAGATTTATTTGGAAGATTGGAGATGTCGACATCGATTCCGAT
AGAAGAGACATTGAGGCAATTGTATGGGAAGATCATGAACCAAAGACTTTTTGGGACAAATTTTGGAATGTTGTAGCATAG

>7 G to T substitution at position 509
ATGACAAATTCAAAAGAAGACGCCGACATAGAGGAGAAGCATATGTACAATGAGCCGGTCACAACCCTCTTTCACGACGTTGAAGCTTCACAAA
CACACCACAGACGTGGGTCAATACCATTGAAAGATGAGAAAAGTAAAGAATTGTATCCATTGCGCTCTTTCCCGACGAGAGTAAATGGCGAGGA
TACGTTCTCTATGGAGGATGGCATAGGTGATGAAGATGAAGGAGAAGTACAGAACGCTGAAGTGAAGAGAGAGCTTAAGCAAAGACATATTGGT
ATGATTGCCCTTGGTGGTACTATTGGTACAGGTCTTTTCATTGGTTTATCCACACCTCTGACCAACGCCGGCCCAGTGGGCGCTCTTATATCAT
ATTTATTTATGGGTTCTTTGGCATATTCTGTCACGCAGTCCTTGGGTGAAATGGCTACATTCATCCCTGTTACATCCTCTTTCACAGTTTTCTC
ACAAAGATTCCTTTCTCCAGCATTTGGTGCGGCCAATGATTACATGTATTGGTTTTCTTGGGCAATCACTTTTGCCCTGGAACTTAGTGTAGTT
GGCCAAGTCATTCAATTTTGGACGTACAAAGTTCCACTGGCGGCATGGATTAGTATTTTTTGGGTAATTATCACAATAATGAACTTGTTCCCTG
TCAAATATTACGGTGAATTCGAGTTCTGGGTCGCTTCCATCAAAGTTTTAGCCATTATCGGGTTTCTAATATACTGTTTTTGTATGGTTTGTGG
TGCTGGGGTTACCGGCCCAGTTGGATTCCGTTATTGGAGAAACCCAGGTGCCTGGGGTCCAGGTATAATATCTAAGGATAAAAACGAAGGGAGG
TTCTTAGGTTGGGTTTCCTCTTTGATTAACGCTGCCTTCACATTTCAAGGTACTGAACTAGTTGGTATCACTGCTGGTGAAGCTGCAAACCCCA
GAAAATCCGTTCCAAGAGCCATCAAAAAAGTTGTTTTCCGTATCTTAACCTTCTACATTGGCTCTCTATTATTCATTGGACTTTTAGTTCCATA
CAATGACCCTAAACTAACACAATCTACTTCCTACGTTTCTACTTCTCCCTTTATTATTGCTATTGAGAACTCTGGTACAAAGGTTTTGCCACAT
ATCTTCAACGCTGTTATCTTAACAACCATTATTTCTGCCGCAAATTCAAATATTTACGTTGGTTCCCGTATTTTATTTGGTCTATCAAAGAACA
AGTTGGCTCCTAAATTCCTGTCAAGGACCACCAAAGGTGGTGTTCCATACATTGCAGTTTTCGTTACTGCTGCATTTGGCGCTTTGGCTTACAT
GGAGACATCTACTGGTGGTGACAAAGTTTTCGAATGGCTATTAAATATCACTGGTGTTGCAGGCTTTTTTGCATGGTTATTTATCTCAATCTCG
CACATCAGATTTATGCAAGCTTTGAAATACCGTGGCATCTCTCGTGACGAGTTACCATTTAAAGCTAAATTAATGCCCGGCTTGGCTTATTATG
CGGCCACATTTATGACGATCATTATCATTATTCAAGGTTTCACGGCTTTTGCACCAAAATTCAATGGTGTTAGCTTTGCTGCCGCCTATATCTC
TATTTTCCTGTTCTTAGCTGTTTGGATCTTATTTCAATGCATATTCAGATGCAGATTTATTTGGAAGATTGGAGATGTCGACATCGATTCCGAT
AGAAGAGACATTGAGGCAATTGTATGGGAAGATCATGAACCAAAGACTTTTTGGGACAAATTTTGGAATGTTGTAGCATAG

>8 Deletion of AAGCA at position 264
ATGACAAATTCAAAAGAAGACGCCGACATAGAGGAGAAGCATATGTACAATGAGCCGGTCACAACCCTCTTTCACGACGTTGAAGCTTCACAAA
CACACCACAGACGTGGGTCAATACCATTGAAAGATGAGAAAAGTAAAGAATTGTATCCATTGCGCTCTTTCCCGACGAGAGTAAATGGCGAGGA
TACGTTCTCTATGGAGGATGGCATAGGTGATGAAGATGAAGGAGAAGTACAGAACGCTGAAGTGAAGAGAGAGCTT_____AAGACATATTGGT
ATGATTGCCCTTGGTGGTACTATTGGTACAGGTCTTTTCATTGGTTTATCCACACCTCTGACCAACGCCGGCCCAGTGGGCGCTCTTATATCAT
ATTTATTTATGGGTTCTTTGGCATATTCTGTCACGCAGTCCTTGGGTGAAATGGCTACATTCATCCCTGTTACATCCTCTTTCACAGTTTTCTC
ACAAAGATTCCTTTCTCCAGCATTTGGTGCGGCCAATGGTTACATGTATTGGTTTTCTTGGGCAATCACTTTTGCCCTGGAACTTAGTGTAGTT
GGCCAAGTCATTCAATTTTGGACGTACAAAGTTCCACTGGCGGCATGGATTAGTATTTTTTGGGTAATTATCACAATAATGAACTTGTTCCCTG
TCAAATATTACGGTGAATTCGAGTTCTGGGTCGCTTCCATCAAAGTTTTAGCCATTATCGGGTTTCTAATATACTGTTTTTGTATGGTTTGTGG
TGCTGGGGTTACCGGCCCAGTTGGATTCCGTTATTGGAGAAACCCAGGTGCCTGGGGTCCAGGTATAATATCTAAGGATAAAAACGAAGGGAGG
TTCTTAGGTTGGGTTTCCTCTTTGATTAACGCTGCCTTCACATTTCAAGGTACTGAACTAGTTGGTATCACTGCTGGTGAAGCTGCAAACCCCA
GAAAATCCGTTCCAAGAGCCATCAAAAAAGTTGTTTTCCGTATCTTAACCTTCTACATTGGCTCTCTATTATTCATTGGACTTTTAGTTCCATA
CAATGACCCTAAACTAACACAATCTACTTCCTACGTTTCTACTTCTCCCTTTATTATTGCTATTGAGAACTCTGGTACAAAGGTTTTGCCACAT
ATCTTCAACGCTGTTATCTTAACAACCATTATTTCTGCCGCAAATTCAAATATTTACGTTGGTTCCCGTATTTTATTTGGTCTATCAAAGAACA
AGTTGGCTCCTAAATTCCTGTCAAGGACCACCAAAGGTGGTGTTCCATACATTGCAGTTTTCGTTACTGCTGCATTTGGCGCTTTGGCTTACAT
GGAGACATCTACTGGTGGTGACAAAGTTTTCGAATGGCTATTAAATATCACTGGTGTTGCAGGCTTTTTTGCATGGTTATTTATCTCAATCTCG
CACATCAGATTTATGCAAGCTTTGAAATACCGTGGCATCTCTCGTGACGAGTTACCATTTAAAGCTAAATTAATGCCCGGCTTGGCTTATTATG
CGGCCACATTTATGACGATCATTATCATTATTCAAGGTTTCACGGCTTTTGCACCAAAATTCAATGGTGTTAGCTTTGCTGCCGCCTATATCTC
TATTTTCCTGTTCTTAGCTGTTTGGATCTTATTTCAATGCATATTCAGATGCAGATTTATTTGGAAGATTGGAGATGTCGACATCGATTCCGAT
AGAAGAGACATTGAGGCAATTGTATGGGAAGATCATGAACCAAAGACTTTTTGGGACAAATTTTGGAATGTTGTAGCATAG

Functional analysis of pVirF-NCasF and pJ, pL, pT-sgRNA-PDS2 in *Agrobacterium*

The pJ-sgRNA-PDS2, pL-sgRNA-PDS2, and pT-sgRNA-PDS2 cassettes were cloned individually into the plasmid expressing NCas9F. Plasmid interference assays were performed to confirm the production of an active NCas9F-sgRNA complex in *Agrobacterium* (Figure S2). *Agrobacterium* cells harboring the plasmid expressing both the sgRNA and NCas9F were transformed with pK2GW8 (pK2-NST) or pK2GW8-NB-PDS (pK2-T) containing the PDS target and streaked on plates with the antibiotics (40mg/l Gm and 100 mg/l Spec) for selection of both plasmids and 0,2 mM acetosyringone for induction of Cas9 expression. Colonies expressing NCas9F and sgRNA-PDS2 and the pK2GW8 plasmid without the PDS target site were selected, whereas no colonies were obtained when selected for the presence of the plasmid containing the PDS target site.

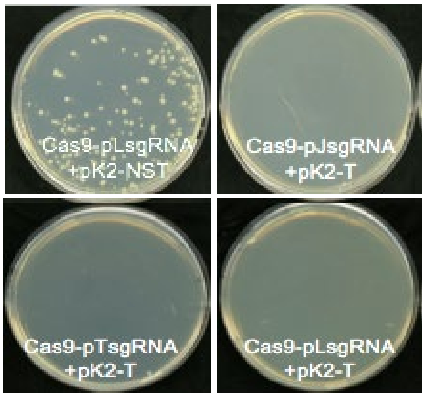

Figure S3 Plasmid loss in *Agrobacterium* strains expressing NCas9F and pL-sgRNA-PDS2, pJ-sgRNA-PDS2 or pT-sgRNA-PDS2 transformed with pK2GW8 (upper left panel) or pK2GW8-NB-PDS containing the PDS2 target (upper right and lower panels).

Primer	Used for	Sequence
DS086	Amplification CAN1 target	CTTCAGACTTCTTAACTCCTGT
DS088	Amplification CAN1 target	TGAGGGTGAGAATGCGAAATG
DS190	Cloning PDS1 sgRNA	ATTGGGCATGCAAAGTCTCTCAGG
DS191	Cloning PDS1 sgRNA	AAACCCTGAGAGACTTTGCATGCC
DS192	Amplification PDS1 target	GGTGTGCCTGATAGGGTGAC
DS193	Amplification PDS1 target	GGCAAACACAAAAGCATCTCC
SP669	PCR1 NGS PDS1 target	CGTGTGCTCTTCCGATCTTCCACTCTGTTTAACTTTTCTG
SP670	PCR1 NGS PDS1 target	GATGTGTATAAGAGACAGCGTGAGTTTAGTCTGACTTG
M30	Amplification PDS2 target	GGCTTAATTTACTGCTATCTTGTTCA
M31	Amplification PDS2 target	CAGCATCACACTTTCGCATT
NBPDS3-gDNA-404bp-F	Amplification PDS2 target	GTAAAATGCCCCAAATTGGACTTGT
NBPDS3-gDNA-404bp-R	Amplification PDS2 target	CGTGAGGAAGTACGAAATGATGATGA

Table S1 Primers used for cloning sgRNA and PCR

	Sample name	DELETION	DELIN	INSERTION	WT	TOTAL	P5	P7
WT	WT[a]_PDS[b]_daan[c]_1[d]	225 (0.05%)	161 (0.04%)	313 (0.07%)	427798 (99.84%)	428497	P5_28	P7_8
P	CasF_PDS_1_1	238	720	110	601135	602203	P5_2	P7_2
	CasF_PDS_3_1	196	418	13	401071	401698	P5_16	P7_16
	CasF_PDS_3_2	312	230	155	475852	476549	P5_17	P7_17
P	combined	746 (0.05%)	1368 (0.09%)	278 (0.02)	1478058 (99.84%)	1480450		
P+T	CasF+T-DNAsg_PDS_daan_1	869	278	3348	421693	426188	P5_29	P7_9
	CasF+T-DNAsg_PDS_daan_2	9019	6216	37931	291855	345021	P5_30	P7_10
	CasF+T-DNAsg_PDS_daan_3	2217	639	8243	626919	638018	P5_16	P7_8
P+T	combined	9888 (0.86%)	6494 (0.51%)	41279 (3,51%)	713548 (95.12%)	771209		
T-DNA	T-DNACassg_PDS_1_1	8670	5814	52575	308432	375491	P5_8	P7_8
	T-DNACassg_PDS_1_2	8052	5520	52210	443312	509094	P5_9	P7_9
	T-DNACassg_PDS_3_1	21810	10771	103705	332841	469127	P5_26	P7_6
	T-DNACassg_PDS_3_2	28465	18382	139130	449105	635082	P5_27	P7_7
	T-DNACassg_PDS_daan_1	17992	9946	67644	295955	391537	P5_31	P7_11
	T-DNACassg_PDS_daan_2	13356	7684	53900	216015	290955	P5_32	P7_12
T-DNA	combined	98345 (3.68%)	58117 (2.18%)	469164 (17.56%)	2045660 (76.58%)	2671286		

Table S2 Samples tested for NGS derive from untransformed tissue (WT) or tissue transformed with NCas9F protein alone (P) or in combination with sgRNA on T-DNA (P+T) or transformed with T-DNA encoding Cas9 and sgRNA (T-DNA). Number and percentages of deletions, insertions, combinations (delin) and wild-type sequences obtained by paired-end sequencing. Barcode primers for each sample are indicated. a: experimental conditions, b: DNA target PDS1, c: experiment name/number, d: sample number.

Primer	Sequence	Barcode
P5_2	AATGATACGGCGACCACCGAGATCTACACagtataacTCGTCGGCAGCGTCAGATGTGTATAAGAGACA*G	AGTATAAC
P5_8	AATGATACGGCGACCACCGAGATCTACACGCAAGCATTCGTCGGCAGCGTCAGATGTGTATAAGAGACA*G	GCAAGCAT
P5_9	AATGATACGGCGACCACCGAGATCTACACGGCATCGATCGTCGGCAGCGTCAGATGTGTATAAGAGACA*G	GGCATCGA
P5_16	AATGATACGGCGACCACCGAGATCTACACGCGTAAGATCGTCGGCAGCGTCAGATGTGTATAAGAGACA*G	GCGTAAGA
P5_17	AATGATACGGCGACCACCGAGATCTACACTATAGCCTTCGTCGGCAGCGTCAGATGTGTATAAGAGACA*G	TATAGCCT
P5_26	AATGATACGGCGACCACCGAGATCTACACGCTAATGGTCGTCGGCAGCGTCAGATGTGTATAAGAGACA*G	GCTAATGG
P5_27	AATGATACGGCGACCACCGAGATCTACACGTAAGTTGTCGTCGGCAGCGTCAGATGTGTATAAGAGACA*G	GTAAGTTG
P5_28	AATGATACGGCGACCACCGAGATCTACACAACGCAATTCGTCGGCAGCGTCAGATGTGTATAAGAGACA*G	AACGCAAT
P5_29	AATGATACGGCGACCACCGAGATCTACACAGATGCCATCGTCGGCAGCGTCAGATGTGTATAAGAGACA*G	AGATGCCA
P5_30	AATGATACGGCGACCACCGAGATCTACACAGCTAGAGTCGTCGGCAGCGTCAGATGTGTATAAGAGACA*G	AGCTAGAG
P5_31	AATGATACGGCGACCACCGAGATCTACACATTAACTGTCGTCGGCAGCGTCAGATGTGTATAAGAGACA*G	ATTAACTG
P5_32	AATGATACGGCGACCACCGAGATCTACACTAGACTGGTCGTCGGCAGCGTCAGATGTGTATAAGAGACA*G	TAGACTGG
P7_2	CAAGCAGAAGACGGCATACGAGATATTACGCTGTGACTGGAGTTCAGACGTGTGCTCTTCCGATC*T	ATTACGCT
P7_6	CAAGCAGAAGACGGCATACGAGATGTTCCTCGGTGACTGGAGTTCAGACGTGTGCTCTTCCGATC*T	GTTCCTCG
P7_7	CAAGCAGAAGACGGCATACGAGATAGCGTAGCGTGACTGGAGTTCAGACGTGTGCTCTTCCGATC*T	AGCGTAGC
P7_8	CAAGCAGAAGACGGCATACGAGATCAGCCTCGGTGACTGGAGTTCAGACGTGTGCTCTTCCGATC*T	CAGCCTCG
P7_9	CAAGCAGAAGACGGCATACGAGATTGCCTCTTGTGACTGGAGTTCAGACGTGTGCTCTTCCGATC*T	TGCCTCTT
P7_10	CAAGCAGAAGACGGCATACGAGATTCCTCTACGTGACTGGAGTTCAGACGTGTGCTCTTCCGATC*T	TCCTCTAC
P7_11	CAAGCAGAAGACGGCATACGAGATAGTTACGTGTGACTGGAGTTCAGACGTGTGCTCTTCCGATC*T	AGTTACGT
P7_12	CAAGCAGAAGACGGCATACGAGATATACGACGGTGACTGGAGTTCAGACGTGTGCTCTTCCGATC*T	ATACGACG
P7_16	CAAGCAGAAGACGGCATACGAGATgccggaacGTGACTGGAGTTCAGACGTGTGCTCTTCCGATC*T	GCCGGAAC
P7_17	CAAGCAGAAGACGGCATACGAGATggaggtccGTGACTGGAGTTCAGACGTGTGCTCTTCCGATC*T	GGAGGTCC

Table S3 Barcode P5 and P7 primers used for NGS.

Sequence of plasmid pNCasF (8620 bp).

AGCTTATCGATACCGTCGATCGACCTCGAGGGGGGGCCCGGTACCCAGCTTTTGTTCCCTTTAGTGAGGGTTAATTGCGCGCTTGGCGTAATCA
TGGTCATAGCTGTTTCCTGTGTGAAATTGTTATCCGCTCACAATTCCACACAACATACGAGCCGGAAGCATAAAGTGTAAAGCCTGGGGTGCCT
AATGAGTGAGCTAACTCACATTAATTGCGTTGCGCTCACTGCCCGCTTTCCAGTCGGGAAACCTGTCGTGCCAGCTGCATTAATGAATCGGCCA
ACGCGCGGGGAGAGGCGGTTTGCGTATTGGGCGCATGGGGTACCGAGCTCGAATTGGCCGCGGCGTTGTGACAATTTACCGAACAACTCCGCGG
CCGGGAAGCCGATCTCGGCTTGAACGAATTGTTAGGTGGCGGTACTTGGGTCGATATCAAAGTGCATCACTTCTTCCCGTATGCCCAACTTTGT
ATAGAGAGCCACTGCGGGATCGTCACCGTAATCTGCTTGCACGTAGATCACATAAGCACCAAGCGCGTTGGCCTCATGCTTGAGGAGATTGATG
AGCGCGGTGGCAATGCCCTGCCTCCGGTGCTCGCCGGAGACTGCGAGATCATAGATATAGATCTCACTACGCGGCTGCTCAAACCTGGGCAGAA
CGTAAGCCGCGAGAGCGCCAACAACCGCTTCTTGGTCGAAGGCAGCAAGCGCGATGAATGTCTTACTACGGAGCAAGTTCCCGAGGTAATCGGA
GTCCGGCTGATGTTGGGAGTAGGTGGCTACGTCTCCGAACTCACGACCGAAAAGATCAAGAGCAGCCCGCATGGATTTGACTTGGTCAGGGCCG
AGCCTACATGTGCGAATGATGCCCATACTTGAGCCACCTAACTTTGTTTTAGGGCGACTGCCCTGCTGCGTAACATCGTTGCTGCTGCGTAACA
TCGTTGCTGCTCCATAACATCAAACATCGACCCACGGCGTAACGCGCTTGCTGCTTGGATGCCCGAGGCATAGACTGTACAAAAAAACAGTCAT
AACAAGCCATGAAAACCGCCACTGCGCCGTTACCACCGCTGCGTTCGGTCAAGGTTCTGGACCAGTTGCGTGAGCGCATACGCTACTTGCATTA
CAGTTTACGAACCGAACAGGCTTATGTCAATTCGAGCTCGGTACCCCGCCCTACGGGCTTGCTCTCCGGGCTTCGCCCTGCGCGGTCGCTGCGC
TCCCTTGCCAGCCCGTGGATATGTGGACGATGGCCGCGAGCGGCCACCGGCTGGCTCGCTTCGCTCGGCCCGTGGACAACCCTGCTGGACAAGC
TGATGGACAGGCTGCGCCTGCCCACGAGCTTGACCACAGGGATTGCCCACCGGCTACCCAGCCTTCGACCACATACCCACCGGCTCCAACTGCG
CGGCCTGCGGCCTTGCCCCATCAATTTTTTTAATTTTCTCTGGGGAAAAGCCTCCGGCCTGCGGCCTGCGCGCTTCGCTTGCCGGTTGGACACC
AAGTGGAAGGCGGGTCAAGGCTCGCGCAGCGACCGCGCAGCGGCTTGGCCTTGACGCGCCTGGAACGACCCAAGCCTATGCGAGTGGGGGCAGT
CGAAGGCGAAGCCCGCCCGCCTGCCCCCCGAGCCTCACGGCGGCGAGTGCGGGGGTTCCAAGGGGGCAGCGCCACCTTGGGCAAGGCCGAAGGC
CGCGCAGTCGATCAACAAGCCCCGGAGGGGCCACTTTTTGCCGGAGGGGGAGCCGCGCCGAAGGCGTGGGGGAACCCCGCAGGGGTGCCCTTCT
TTGGGCACCAAAGAACTAGATATAGGGCGAAATGCGAAAGACTTAAAAATCAACAACTTAAAAAAGGGGGGTACGCAACAGCTCATTGCGGCAC
CCCCCGCAATAGCTCATTGCGTAGGTTAAAGAAAATCTGTAATTGACTGCCACTTTTACGCAACGCATAATTGTTGTCGCGCTGCCGAAAAGTT
GCAGCTGATTGCGCATGGTGCCGCAACCGTGCGGCACCCTACCGCATGGAGATAAGCATGGCCACGCAGTCCAGAGAAATCGGCATTCAAGCCA
AGAACAAGCCCGGTCACTGGGTGCAAACGGAACGCAAAGCGCATGAGGCGTGGGCCGGGCTTATTGCGAGGAAACCCACGGCGGCAATGCTGCT
GCATCACCTCGTGGCGCAGATGGGCCACCAGAACGCCGTGGTGGTCAGCCAGAAGACACTTTCCAAGCTCATCGGACGTTCTTTGCGGACGGTC
CAATACGCAGTCAAGGACTTGGTGGCCGAGCGCTGGATCTCCGTCGTGAAGCTCAACGGCCCCGGCACCGTGTCGGCCTACGTGGTCAATGACC
GCGTGGCGTGGGGCCAGCCCCGCGACCAGTTGCGCCTGTCGGTGTTCAGTGCCGCCGTGGTGGTTGATCACGACGACCAGGACGAATCGCTGTT
GGGGCATGGCGACCTGCGCCGCATCCCGACCCTGTATCCGGGCGAGCAGCAACTACCGACCGGCCCCGGCGAGGAGCCGCCCAGCCAGCCCGGC
ATTCCGGGCATGGAACCAGACCTGCCAGCCTTGACCGAAACGGAGGAATGGGAACGGCGCGGGCAGCAGCGCCTGCCGATGCCCGATGAGCCGT
GTTTTCTGGACGATGGCGAGCCGTTGGAGCCGCCGACACGGGTCACGCTGCCGCGCCGGTAGCACTTGGGTTGCGCAGCAACCCGTAAGTGCGC
TGTTCCAGACTATCGGCTGTAGCCGCCTCGCCGCCCTATACCTTGTCTGCCTCCCCGCGTTGCGTCGCGGTGCATGGAGCCGGGCCACCTCGAC
CTGAATGGAAGCCGGCGGCACCTCGCTAACGGATTCACCGTTTTTATCAGGCTCTGGGAGGCAGAATAAATGATCATATCGTCAATTATTACCT
CCACGGGGAGAGCCTGAGCAAACTGGCCTCAGGCATTTGAGAAGCACACGGTCACACTGCTTCCGGTAGTCAATAAACCGGTAAACCAGCAATA
GACATAAGCGGCTATTTAACGACCCTGCCCTGAACCGACGACCGGGTCGAATTTGCTTTCGAATTTCTGCCATTCATCCGCTTATTATCACTTA
TTCAGGCGTAGCACCAGGCGTTTAAGGGCACCAATAACTGCCTTAAAAAAATTACGCCCCGCCCTGCCACTCATCGCAGTCGGCCTATTGGTTA
AAAAATGAGCTGATTTAACAAAAATTTAACGCGAATTTTAACAAAATATTAACGCTTACAATTTCCATTCGCCATTCAGGCTGCGCAACTGTTG
GGAAGGGCGATCGGTGCGGGCCTCTTCGCTATTACGCCAGCTGGCGAAAGGGGGATGTGCTGCAAGGCGATTAAGTTGGGTAACGCCAGGGTTT
TCCCAGTCACGACGTTGTAAAACGACGGCCAGTGAGCGCGCGTAATACGACTCACTATAGGGCGAATTGGAGCTCCACCGCGGTGGCGGCCGCA
CCTAAAGACCGGGGCAGTTGAAATCCGATTGTGATGGCAACTATAAGCGGGTGACGAACAGCACGGATAGTCATAGACCGCGCGTTGATCGAGG
TCTGTCCGCCGACATTAATTCCGCTCGCGCATCGTCGTGAGCCGTTTTAATGGATCGGGCTATGGGCCGAACTTCTGCCATAACCTCGAGTCGA
CCACTAGTAGATCCGCTGCTGCCGTTGCTCCCACTACCAATGCCGTCCACTTTCCGCTTTTTCTTAGGATCTTCCACCTTGCGTTTTTTCTTGG
GGGAGCCGCTGCCCAGGCTGGCATAATCGGGCACGTCATAGGGATAGGCGTCGCCTCCCAGCTGAGACAGGTCGATCCGTGTCTCGTACAGGCC
GGTGATGCTCTGGTGGATCAGGGTGGCGTCCAGCACCTCTTTGGTGCTGGTGTACCTCTTCCGGTCGATGGTGGTGTCAAAGTACTTGAAGGCG
GCAGGGGCTCCCAGATTGGTCAGGGTAAACAGGTGGATGATATTCTCGGCCTGCTCTCTGATAGGCTTGTCTCTGTGCTTGTTGTAGGCGCTCA
GCACCTTGTCCAGATTAGCGTCGGCCAGGATCACTCTCTTGGAGAACTCGCTGATCTGCTCGATGATCTCGTCCAGGTAGTGTTTGTGCTGTTC
CACAAACAGCTGTTTCTGCTCATTATCCTCGGGGGAGCCCTTCAGCTTCTCATAGTGGCTGGCCAGGTACAGGAAGTTCACATATTTGGAGGGC
AGGGCCAGTTCGTTTCCCTTCTGCAGTTCGCCGGCAGAGGCCAGCATTCTCTTCCGGCCGTTTTCCAGCTCGAACAGGGAGTACTTAGGCAGCT
TGATGATCAGGTCCTTTTTCACTTCTTTGTAGCCCTTGGCTTCCAGAAAGTCGATGGGATTCTTCTCGAAGCTGCTTCTTTCCATGATGGTGAT
CCCCAGCAGCTCTTTCACACTCTTCAGTTTCTTGGACTTGCCCTTTTCCACTTTGGCCACCACCAGCACAGAATAGGCCACGGTGGGGCTGTCG
AAGCCGCCGTACTTCTTAGGGTCCCAGTCCTTCTTTCTGGCGATCAGCTTGTCGCTGTTCCTCTTGGGCAGGATAGACTCTTTGCTGAAGCCGC
CTGTCTGCACCTCGGTCTTTTTCACGATATTCACTTGGGGCATAGACAGCACTTTCCGCACGGTGGCAAAGTCCCGGCCCTTATCCCACACGAT
CTCGCCTGTTTCGCCGTTTGTCTCGATCAGAGGCCGCTTCCGGATCTCGCCGTTGGCCAGGGTAATCTCGGTCTTGAAAAAGTTCATGATGTTG
CTGTAGAAGAAGTACTTGGCGGTAGCCTTGCCGATTTCCTGCTCGCTCTTGGCGATCATCTTCCGCACGTCGTACACCTTGTAGTCGCCGTACA
CGAACTCGCTTTCCAGCTTAGGGTACTTTTTGATCAGGGCGGTTCCCACGACGGCGTTCAGGTAGGCGTCGTGGGCGTGGTGGTAGTTGTTGAT
CTCGCGCACTTTGTAAAACTGGAAATCCTTCCGGAAATCGGACACCAGCTTGGACTTCAGGGTGATCACTTTCACTTCCCGGATCAGTTTGTCG
TTCTCGTCGTACTTAGTGTTCATCCGGGAGTCCAGGATCTGTGCCACGTGCTTTGTGATCTGCCGGGTTTCCACCAGCTGTCTCTTGATGAAGC
CGGCCTTATCCAGTTCGCTCAGGCCGCCTCTCTCGGCCTTGGTCAGATTGTCGAACTTCCTCTGGGTAATCAGCTTGGCATTCAGCAGCTGGCG
CCAGTAGTTCTTCATCTTCTTCACGACCTCTTCGGAGGGCACGTTGTCGCTCTTGCCCCGGTTCTTGTCGCTCCGAGTCAGCACTTTGTTATCG
ATGGAGTCGTCCTTCAGAAAGCTCTGAGGCACGATATGGTCCACATCGTAGTCGGACAGCCGGTTGATGTCCAGTTCCTGGTCCACGTACATAT
CCCGCCCATTCTGCAGGTAGTACAGGTACAGCTTCTCGTTCTGCAGCTGGGTGTTTTCCACGGGGTGTTCTTTCAGGATCTGGCTGCCCAGCTC
TTTGATGCCCTCTTCGATCCGCTTCATTCTCTCGCGGCTGTTCTTCTGTCCCTTCTGGGTGGTCTGGTTCTCTCTGGCCATTTCGATCACGATG
TTCTCGGGCTTGTGCCGGCCCATCACTTTCACGAGCTCGTCCACCACCTTCACTGTCTGCAGGATGCCCTTCTTAATGGCGGGGCTGCCGGCCA
GATTGGCAATGTGCTCGTGCAGGCTATCGCCCTGGCCGGACACCTGGGCTTTCTGGATGTCCTCTTTAAAGGTCAGGCTGTCGTCGTGGATCAG
CTGCATGAAGTTTCTGTTGGCGAAGCCGTCGGACTTCAGGAAATCCAGGATTGTCTTGCCGGACTGCTTGTCCCGGATGCCGTTGATCAGCTTC
CGGCTCAGCCTGCCCCAGCCGGTGTATCTCCGCCGCTTCAGCTGCTTCATCACTTTGTCGTCGAACAGGTGGGCATAGGTTTTCAGCCGTTCCT
CGATCATCTCTCTGTCCTCAAACAGTGTCAGGGTCAGCACGATATCTTCCAGAATGTCCTCGTTTTCCTCATTGTCCAGGAAGTCCTTGTCCTT
GATAATTTTCAGCAGATCGTGGTATGTGCCCAGGGAGGCGTTGAACCGATCTTCCACGCCGGAGATTTCCACGGAGTCGAAGCACTCGATTTTC
TTGAAGTAGTCCTCTTTCAGCTGCTTCACGGTCACTTTCCGGTTGGTCTTGAACAGCAGGTCCACGATGGCTTTTTTCTGCTCGCCGCTCAGGA
AGGCGGGCTTTCTCATTCCCTCGGTCACGTATTTCACTTTGGTCAGCTCGTTGTACACGGTGAAGTACTCGTACAGCAGGCTGTGCTTGGGCAG
CACCTTCTCGTTGGGCAGGTTCTTATCGAAGTTGGTCATCCGCTCGATGAAGCTCTGGGCGCTGGCGCCCTTGTCCACCACTTCCTCGAAGTTC
CAGGGGGTGATGGTTTCCTCGCTCTTTCTGGTCATCCAGGCGAATCTGCTGTTTCCCCTGGCCAGAGGGCCCACGTAGTAGGGGATGCGGAAGG
TCAGGATCTTCTCGATCTTTTCCCGGTTGTCCTTCAGGAATGGGTAAAAATCTTCCTGCCGCCGCAGAATGGCGTGCAGCTCTCCCAGGTGGAT
CTGGTGGGGGATGCTGCCGTTGTCGAAGGTCCGCTGCTTCCGCAGCAGGTCCTCTCTGTTCAGCTTCACGAGCAGTTCCTCGGTGCCGTCCATC
TTTTCCAGGATGGGCTTGATGAACTTGTAGAACTCTTCCTGGCTGGCTCCGCCATCGATGTAGCCGGCGTAGCCGTTCTTGCTCTGGTCGAAGA
AAATCTCTTTGTACTTCTCAGGCAGCTGCTGCCGCACGAGAGCTTTCAGCAGGGTCAGGTCCTGGTGGTGCTCGTCGTATCTCTTGATCATAGA

GGCGCTCAGGGGGGCCTTGGTGATCTCGGTGTTCACTCTCAGGATGTCGCTCAGCAGGATGGCGTCGGACAGGTTCTTGGCGGCCAGAAACAGG
TCGGCGTACTGGTCGCCGATCTGGGCCAGCAGGTTGTCCAGGTCGTCGTCGTAGGTGTCCTTGCTCAGCTGCAGTTTGGCATCCTCGGCCAGGT
CGAAGTTGCTCTTGAAGTTGGGGGTCAGGCCCAGGCTCAGGGCAATCAGGTTGCCGAACAGGCCATTCTTCTTCTCGCCGGGCAGCTGGGCGAT
CAGATTTTCCAGCCGTCTGCTCTTGCTCAGTCTGGCAGACAGGATGGCCTTGGCGTCCACGCCGCTGGCGTTGATGGGGTTTTCCTCGAACAGC
TGGTTGTAGGTCTGCACCAGCTGGATGAACAGCTTGTCCACGTCGCTGTTGTCGGGGTTCAGGTCGCCCTCGATCAGGAAGTGGCCCCGGAACT
TGATCATGTGGGCCAGGGCCAGATAGATCAGCCGCAGGTCGGCCTTGTCGGTGCTGTCCACCAGTTTCTTTCTCAGGTGGTAGATGGTGGGGTA
CTTCTCGTGGTAGGCCACCTCGTCCACGATGTTGCCGAAGATGGGGTGCCGCTCGTGCTTCTTATCCTCTTCCACCAGGAAGGACTCTTCCAGT
CTGTGGAAGAAGCTGTCGTCCACCTTGGCCATCTCGTTGCTGAAGATCTCTTGCAGATAGCAGATCCGGTTCTTCCGTCTGGTGTATCTTCTTC
TGGCGGTTCTCTTCAGCCGGGTGGCCTCGGCTGTTTCTCCGCTGTCGAACAGCAGGGCGCCGATCAGGTTCTTCTTGATGCTGTGCCGGTCGGT
GTTGCCCAGCACCTTGAATTTCTTGCTGGGCACCTTGTACTCGTCGGTGATCACGGCCCAGCCCACAGAGTTGGTGCCGATGTCCAGGCCGATG
CTGTACTTCTTGTCCATGGTGGCATTGGATTGGAAGTAGGGATCTCGACCGGTACCCAATTCGACCTTTCTCTTCTTTTTTGGAGGCTCGGGAA
TTAATTCCGCTTTATCCATGATATCGCTCCTGTGCTTTTGAAAGGATGATATGCTACGCGGGTGCAGATTATTAAGAAGTTTTCAGTTTCATTT
GTAGTTGTGTGGCAAAATAGATCAGCTTCGGCATCGGCAAAGCGTTTTGCTGGCAATCTTTCGGCGCAGCCGAGAAAAAGGTTACTGGTTGCGA
ATACTCAAAAGGAAAAACCTACCTGCATTAGTCAGTGCCGCTTTTATTCTCTTACGATCAGTCATTAACGGACACCGAAGCCACCGCCCAAAGC
ACCAGCCTGGGCCAAAGATCGGTGGCATAGAAGTTGGCCACGCCCTGTGGCAAACCGCGGCAGGCCGCCAGGAAATAGCCCACCGCAACAAGTG
TGGCGATCGTACGTGACGGCGCCATTGCCCAGAGCAAAGGAAAGAACATGGCGGGTGGAAGAAGAAGCACCTCCCCGCTCCAGCCGATCCACCC
ACAGGCGATGGATGCGATGATCAGGAGCACGGATTTCCAGCGATCAGCCAGATGATCGTCACCGCGATCACGGGTGGAAGGTGAACACGGGCCG
CGCCAGGCTGAGAAGACCGGAATCCGGAACCGGACCAAAATATCGACTATCATAGGAGCTCGCGAA

Sequence of pJ-NB-PDS-sgRNA.

GGTGGCggccgcGACGACCGGGTCTCTAGAgAATTCTAAAGATCTTTGACAGCTAGCTCAGTCCTAGGTATAATACTAGTTTGGTAGTAGCGAC
TCCATGGTTTTAGAGCTAGAAATAGCAAGTTAAAATAAGGCTAGTCCGTTATCAACTTGAAAAAGTGGCACCGAGTCGGTGCTTTTTTTGAAGC
TTGGGCCCGAACAAAAACTCatctcagaagaGGATCCTTCGAAgcGGCCG

Sequence of pT-NB-PDS-sgRNA.

GGTGGCggccgcGACGACCGGGTCTCTAGAgAATTCTAAAGATCTGTATTAAGTATTGTTTTATGGCTGATAAATTTCTTTGAATTTCTCCTTG
ATTATTTGTTATAAAAGTTATAAAATAATCTTGTTTTGGTAGTAGCGACTCCATGGTTTTAGAGCTAGAAATAGCAAGTTAAAATAAGGCTAGT
CCGTTATCAACTTGAAAAAGTGGCACCGAGTCGGTGCTTTTTTTGAAGCTTGGGCCCGAACAAAAACTCatctcagaagaGGATCCTTCGAAgc
GGCCG

Sequence of pL-NB-PDS-sgRNA.

GGTGGCggccgcGACGACCGGGTCTCTAGAgAATTCTAAAGATCTATACTCTTAATAAATGCAGTAATACAGGGGCTTTTCAAGACTGAAGTCT
AGCTGAGACAAATAGTGCGATTACAAAATTTTTTAGACAAAAATAGTCTACGAGTTGGTAGTAGCGACTCCATGGTTTTAGAGCTAGAAATAGC
AAGTTAAAATAAGGCTAGTCCGTTATCAACTTGAAAAAGTGGCACCGAGTCGGTGCTTTTTTTGAAGCTTGGGCCCGAACAAAAACTCatctca
gaagaGGATCCTTCGAAgcGGCCG

Protospacer is shown in yellow.

NGS data can be found at:

http://www.ncbi.nlm.nih.gov/bioproject/622227

http://www.ncbi.nlm.nih.gov/bioproject/634985
